# Substitutions of PrP N-terminal histidine residues modulate scrapie disease pathogenesis and incubation time in transgenic mice

**DOI:** 10.1371/journal.pone.0188989

**Published:** 2017-12-08

**Authors:** Sabina Eigenbrod, Petra Frick, Uwe Bertsch, Gerda Mitteregger-Kretzschmar, Janina Mielke, Marko Maringer, Niklas Piening, Alexander Hepp, Nathalie Daude, Otto Windl, Johannes Levin, Armin Giese, Vignesh Sakthivelu, Jörg Tatzelt, Hans Kretzschmar, David Westaway

**Affiliations:** 1 Center for Neuropathology and Prion Research, Ludwig Maximilians University, Munich, Germany; 2 Centre for Prions and Protein Folding Diseases, University of Alberta, Edmonton, Alberta, Canada; 3 Department of Metabolic Biochemistry/Neurobiochemistry, Adolf Butenandt Institute, Ludwig Maximilians University, Munich, Germany; IRCCS - Mario Negri Institute for Pharmacological Research, ITALY

## Abstract

Prion diseases have been linked to impaired copper homeostasis and copper induced-oxidative damage to the brain. Divalent metal ions, such as Cu^2+^ and Zn^2+^, bind to cellular prion protein (PrP^C^) at octapeptide repeat (OR) and non-OR sites within the N-terminal half of the protein but information on the impact of such binding on conversion to the misfolded isoform often derives from studies using either OR and non-OR peptides or bacterially-expressed recombinant PrP. Here we created new transgenic mouse lines expressing PrP with disrupted copper binding sites within all four histidine-containing OR's (sites 1–4, H60G, H68G, H76G, H84G, "TetraH>G" allele) or at site 5 (composed of residues His-95 and His-110; "H95G" allele) and monitored the formation of misfolded PrP *in vivo*. Novel transgenic mice expressing PrP(TetraH>G) at levels comparable to wild-type (wt) controls were susceptible to mouse-adapted scrapie strain RML but showed significantly prolonged incubation times. In contrast, amino acid replacement at residue 95 accelerated disease progression in corresponding PrP(H95G) mice. Neuropathological lesions in terminally ill transgenic mice were similar to scrapie-infected wt controls, but less severe. The pattern of PrP^Sc^ deposition, however, was not synaptic as seen in wt animals, but instead dense globular plaque-like accumulations of PrP^Sc^ in TgPrP(TetraH>G) mice and diffuse PrP^Sc^ deposition in (TgPrP(H95G) mice), were observed throughout all brain sections. We conclude that OR and site 5 histidine substitutions have divergent phenotypic impacts and that *cis* interactions between the OR region and the site 5 region modulate pathogenic outcomes by affecting the PrP globular domain.

## Introduction

Prion diseases such as Creutzfeldt-Jakob disease and bovine spongiform encephalopathy are progressive neurodegenerative disorders that are caused by the structural conversion and aggregation of the normal cellular prion protein (PrP^C^) to a misfolded, partially protease-resistant isoform (PrP^Sc^) [1]. Structurally, PrP^C^ consists of a globular folded C-terminal domain, which is largely α-helical, and a glycine-rich N-terminal domain containing an octapeptide repeat region (OR) composed of four contiguous copies of the highly conserved PHGGGWGQ sequence [2]. The mechanistic details of replication and disease pathogenesis are not understood in detail, but appear to involve direct interaction between PrP^C^ and PrP^Sc^ [3, 4]. In this context, the intrinsically disordered N-terminal domain of PrP^C^ is suggested to play a role in modulating the physiological function of PrP^C^ as well as altering pathological outcomes in prion diseases [1, 5–7]. A multitude of studies demonstrates that the N-terminus of PrP can interact with a broad range of ligands, such as β-sheet-rich conformers (in particular, PrP^Sc^ and Alzheimer’s Disease Aβ42 peptide), metal ions (such as Cu^2+^ and Zn^2+^), lipids, nucleic acids, and glycosaminoglycans, which confer to the protein diverse, and sometimes contrasting, activities, including protection from oxidative stress and intermediation of the toxic effects of PrP^Sc^ (reviewed in [8]). At physiological pH, the N-terminal domain can selectively bind up to six Cu^2+^ ions with the first 2 molar equivalents of Cu^2+^ binding to the amyloidogenic region of human PrP^C^ containing His-96 and His-111 (“site 5”), and then to the four binding sites provided by the OR region [8, 9].

Despite numerous studies, it is unclear whether copper binding to PrP promotes or prevents prion diseases. It was found earlier, that the presence of copper greatly slows amyloid formation [10] and that the addition of copper reduces the accumulation of PrP^Sc^ [11]. Moreover, under physiologically-relevant low Cu^2+^ levels, binding of copper to the OR region enhances the structural stability of the whole N-terminal domain making it more resistant to misfolding [12]. These findings suggest a protective role for copper at the initial stages of prion diseases when bound in the low-occupancy mode. On the other hand, reduction of copper levels in the brain by chelation attenuates the onset of prion disease [13].

More recent findings suggest that distinct redox activities of a copper-bound PrP are involved in the mechanisms underlying prion diseases [14]. In particular, the histidine-rich copper-binding complex derived from the OR region has been shown to exhibit a high reduction potential for the Cu(II)/Cu(I) couple and tends to stabilize the lower-valent Cu(I) state, which can initiate reactions leading to reactive oxygen species (ROS)-mediated events, including β-cleavage [15–19]. Following ROS-mediated β-cleavage at Gly90, further Cu^2+^ coordination to the potential copper binding sites His96 and His111 in human PrP (equivalent to mouse residues 95, 110) can induce localized β-sheet secondary structure, which may subsequently proceed into the C-terminal domain [20–22]. The copper-binding site 5 around His96 in turn exhibits a high oxidation potential for the Cu(II)/Cu(III) couple and stabilizes the higher-valent Cu(III) state which is involved in early events that initiate the misfolding pathway. Notably, alterations of the number of octapeptide repeats [23, 24] have been shown to abrogate the Cu^2+^-dependent endocytosis of PrP, indicating that, although there are non-octarepeat Cu^2+^-binding sites in PrP^C^, it is binding of the metal at the octapeptide repeats that is essential for its internalization. Thus, loss of Cu^2+^-mediated endocytosis of PrP^C^ may also contribute to the disease phenotype.

Information on the role of metal ion binding to PrP and its impact on the conversion to the aberrant isoform is skewed towards studies utilizing either peptides corresponding to the octarepeats or bacterially-expressed recombinant PrP (rPrP). Based on the concept that the N-terminal region of metal-free PrP^C^ is natively unstructured but that different stoichiometries of metal binding can impart different structures upon the OR [25], Lau et al. recently created new PrP alleles which either lock the OR in an extended conformation conductive to binding up to 4 copper ions per OR or in a compact conformation that can only bind one copper ion per OR [26] to restrict inter-conversion and study their biological properties individually. In this study, we altered the OR and binding sites 1–5 in a different way, by substituting the histidine residues with glycine in order to prevent loading of metal ions ("metallation"), and monitored the formation of misfolded PrP *in vivo* in these newly generated transgenic mice.

## Material and methods

### Generation of transgenic mice

We used a derivative of the half-genomic vector phg-*Prnp* [5, 27] designated phg-Sce1 wherein a 3000 bp *Knp*I-*Nar*I-fragment, which contains the entire *Prnp* ORF and part of the 3’-UTR was modified by using by a synthetic double-stranded I-*Sce*I recognition sequence derived from the complementary primer pair 5’-CGCCTAGGGATAACAGGGTAATGTAC-3’ and 5’-ATTACCCTGTTATCCCTA-GG-3’ (a gift from H. Jacobsen). *Xho*I-*Xba*I-fragments from pCi-PrP-TetraH>G and pCi-PrP-H95G, respectively [28], comprising the mutant *Prnp* ORFs were treated with T4-DNA polymerase to fill in 5’-overhangs at both ends and ligated into the phg-Sce-I vector, which had been cleaved with I-*Sce*I and treated with S1-nuclease to remove 3’-overhanging nucleotides. From these plasmids, called phg-PrP-TetraH>G and phg-PrP-H95G, respectively, a *Not*I-*Sal*I fragment was injected into the male pronucleus of fertilized oocytes. Blastulae were transferred to the uteri of foster mothers according to standard techniques. This procedure and the initial screening of tail biopsies of the offspring for the integration of the desired transgene were performed at RCC Ltd (Füllinsdorf, Switzerland). To screen founders, genomic DNA was extracted from tail biopsies and used as template for PCR reactions employing primer pair Exon2 (5’-caaccgagctgaagcattctgcct-3’) and Mut217 (5’-cctgggactccttctggtactgggtgacgc-3’). Transgenic animals were characterized by the generation of a 700 bp fragment during these reactions. Founders generated on a 129Sv genetic background were identified and crossed back into PrP^-/-^ mice (C57BL/6J × 129/Sv genetic background; [28]) to yield *Prnp*^0/0^ lines homozygous for the PrP-encoding transgene array. In doing so, one *Prnp*^0/0^ lines homozygous for the PrP(TetraH>G)-encoding transgene array, designated lines 34, and three *Prnp*^0/0^ lines homozygous for the PrP(H95G)-encoding transgene array, termed lines 4, 11, and 13, respectively, were identified.

### Intracerebral inoculations and scrapie diagnosis

Groups of eight mice each comprising PrP(TetraH>G)^+/+^ mice (lines 34) as well as PrP(H95G)^+/+^ mice of three different founders on PrP^-/-^ background (lines 4, 11, and 13) and wild-type mice (C57BL/6J × 129Sv genetic background), respectively, were intracerebrally (*i*.*c*.) inoculated with mouse-adapted scrapie strain RML (NIH Rocky Mountain Laboratory, Hamilton, MT) using a dose of 30 μl of 10% (w/v) brain homogenate in sterile phosphate buffered saline (PBS). In parallel a 10% (w/v) brain homogenate from uninfected control mice ("mock" homogenate) was used. Inoculation was performed with a Luer lock system (catalog #807.001C; Robert Helwig, Berlin, Germany), and diethylether was used as an anesthetic. At that time the experiments were performed, the use of diethylether to anesthetize mice followed the animal protection regulations at the Ludwig Maximilians University but we note that ether is not any longer advised for use in this role due to potential adverse effects. All animals were kept under standard diurnal pathogen-free conditions and allowed access to food and water *ad libitum*. Mice were monitored every second day for the onset of clinical symptoms, and TSE was diagnosed according to standard clinical criteria used to identify animals exhibiting signs of scrapie, including ataxia, kyphosis, and hind leg paresis [29, 30]. Mice were euthanized at the onset of clinical signs associated with terminal prion disease and extracted brain tissues were subjected to histopathological analyses. The animal experiments were performed in accordance with animal protection standards and were approved by the government of Upper Bavaria (protocol number 209.1-2531-31/02). Every effort was made to reduce the number of animals used and to ensure they were free of pain and discomfort.

For secondary passage 30 μl of 10% (w/v) brain homogenate derived from terminally-diseased PrP(TetraH>G)^+/+^ mice (line 34, mouse #258 and #260, respectively) primarily inoculated with RML prions were *i*.*c*. injected in healthy C57BL/6J 129Sv and PrP(TetraH>G)^+/+^ mice (n = 8 each). In a parallel experiment, 30 μl of 10% brain homogenate derived from two terminally-diseased RML-infected PrPH95G^+/+^ mice (line 13, mouse #304 and #205) were *i*.*c*. inoculated in healthy wild-type and PrP(H95G)^+/+^ mice, respectively.

### Histopathology and immunohistochemistry

Brains from terminally diseased mice were fixed with 4% buffered formalin, subsequently inactivated with 100% formic acid for 1 h, paraffin-embedded and then subjected to routine staining procedures using a Benchmark staining machine with a standard 3’,3-diaminobenzidine (DAB) detection system according to the manufacturer’s instructions (Ventana Medical Systems, Arizona, USA). For detection of PrP^Sc^ monoclonal CDC1 antibody raised against full-length mouse PrP was used (1: 500 in PBS-Tween). Spongiform changes and gliosis were visualized using Hematoxylin and Eosin staining and anti-GFAP polyclonal antiserum (1:1600, DAKO, Germany), respectively. Histological sections were scored in a blinded fashion for spongiform changes in nine standard grey matter areas and three white matter areas as described earlier [31, 32] and lesion profiles were constructed [33, 34]. Scoring of pathological change was performed in the same regions using the following score: 0 = “not detectable”, 1 =“mild”, 2 =“medium”, 3 =“high”, for levels of gliosis or PrP^Sc^ deposition, respectively.

### Histoblotting

Paraffin-embedded-tissue (PET)-blots were prepared as described [35]. In brief, formic acid-decontaminated tissue samples were embedded in paraffin. Thereafter, 4–7 μm paraffin sections were mounted on prewetted 0.45 μm nitrocellulose membrane (Bio-Rad, Munich, Germany). Protease-resistant PrP^Sc^ (after digestion with 250 μg/ml proteinase K overnight at 55°C) and total PrP (no proteinase K digestion) were detected using monoclonal CDC1 antibody (1:2000) and goat anti-rabbit secondary antibody (1:1000). Visualization was performed using 4-nitroblue tetrazolium chloride (NBT) and 5-bromo-4-chloro-3-indolyl phosphate (BCIP) (Boehringer Mannheim, Germany).

### Determination of PrP^Sc^ by western blotting

Brain homogenates were made to 10% (w/v) with lysis buffer (100 mM NaCl, 10 mM EDTA, 0.5% Nonidet P-40, 0.5% sodium deoxycholate, 10 mM Tris, pH 7.6) using a Dounce homogenizer (Schuett-biotec GmbH, Göttingen, Germany). Samples were treated with proteinase K (1 μg/ 10 μl lysate) at 37°C for 30 min and/or PNGase F (New England BioLabs, Frankfurt, Germany) according to the manufacturer´s protocol. Digestion was terminated by boiling in Laemmli sample buffer. Proteins were size-fractionated on a sodium dodecyl sulphate-15% polyacrylamide gel (SDS-PAGE) and transferred to Immobilon-P nitrocellulose membranes (0.2 μm; Millipore, Schwalbach, Germany) by semidry blotting. Membranes were blocked with 5% non-fat dry milk in PBS-T (PBS containing 0.1% Tween-20) for 2 h at room temperature and subsequently incubated with monoclonal antibody 4H11 [36], 6H4 (Prionics, Schlieren-Zurich, Switzerland) or SHA31 (Medicorp, Montreal, QC, Canada) overnight at 4°C. Following washing with PBS-Tween (PBS-T), the membranes were incubated with horseradish peroxidase-linked goat anti-mouse IgG (1:3000; GE Healthcare, Freiburg, Germany) for 1 h at room temperature. After washing the membranes again with PBS-T and incubating them with alkaline phosphatase buffer (100 mM NaCl, 5 mM MgCl_2_, 100 mM Tris; pH 9.5) for 10 min at room temperature, the antigen was detected with CPD-Star (Roche, Mannheim, Germany). Quantification was performed using AIDA 3.26 image analysis software (Raytest, Straubenhardt, Germany).

### Cell-free conversion of ^35^S-labelled PrP^C^

Adherent rabbit kidney epithelial (RK13) cells [37] were co-transfected with vectors pcDNA3.1/Hygro(+) (Invitrogen) and pCI-neo (Promega) carrying either full-length mouse PrP or PrP(TetraH>G) as described previously [38]. Single cell clones resistant to hygromycin B (500 μg/ml, Roche) were expanded and the level of PrP^C^ expression was monitored by western blot analysis after SDS-PAGE of cell lysates.

Labelling of PrP^C^ with ^35^S and purification of PrP^C^ from cell lysates was performed as described previously [38]. Briefly, cell clones were incubated for 1 h at 37°C with 5% CO_2_ in culture medium lacking cysteine and methionine. Tunicamycin was also added in a concentration of 27 μg/ml in order to obtain deglycosylated PrP^C^. After 1 h starvation, Redivue-Promix (GE Healthcare, Munich, Germany) containing ^35^S-methionine and ^35^S-cysteine was added at 0.23 mCi/ml. Cells were lysed after 5 h incubation at 37°C with 5% CO_2_. Lysates were centrifuged for 5 min at 4°C at 1000 × g, the supernatant precipitated with four volumes of cold methanol and the pellet resuspended into a detergent lipid protein complex (DLPC) buffer (150 mM NaCl, 50 mM Tris/HCl pH 8.0, 2% N-laurylsarcosine, 0.4% L-α-phosphatidylcholine) by sonicating three times for 20 s with 70% output intensity using the ultrasound-generator Sonoplus HD2200-UW2200 with BR30 cup-horn sonicator (Bandelin, Berlin, Germany).

PrP^C^ was purified from the DLPC suspension by immunoprecipitation as described by Caughey et al. [39] using the antibody RA3153 [40]. PrP^C^ was eluted from the protein-A sepharose beads with 0.1 M acetic acid, transferred into 1.5 ml low-binding tubes (Eppendorf, Hamburg, Germany) and stored at 4°C. The activity of purified PrP^C^ samples was measured using the β-counter TRI-CARB 2900TR (Perkin Elmer, Rodgau, Germany). Purification of PrP^Sc^ was performed as described previously [41]. The final pellet was resuspended in phosphate buffered saline (PBS) with 0.5% zwittergent sulfobetaine 3–14 (100 μl/2 g brain tissue) by sonication. The resulting suspension was transferred into 1.5 ml low-binding tubes (Eppendorf, Hamburg, Germany) and stored at 4°C. PrP^Sc^ was purified from brains of terminally-diseased *tga*20 mice infected with RML.

*In vitro* conversion reactions with purified PrP^Sc^ and ^35^S-labeled PrP^C^ were performed in a reaction volume of 30 μl as described previously [38]. Briefly, 15,000 cpm ^35^S-PrP^C^ was incubated for 3 days at 37°C with 4 μl of PrP^Sc^ in conversion buffer (200 mM KCl, 5 mM MgCl_2_, 0.625% N-laurylsarcosine, 50 mM sodium citrate pH 6.0) including 0.7 M guanidine hydrochloride (GndHCl). 90% of the reaction volume was digested with proteinase K (PK) for 1 h at 37°C (20 μg/ml) and the remaining 10% was left untreated. Further sample preparation was performed as described [39]. After electrophoresis of untreated and PK-digested samples gels were incubated in fixing solution (isopropanol, H_2_O and acetic acid in a ratio (v/v) of 25:65:10, respectively) for 30 min and subsequently incubated in Amplify (Amersham Biosciences, Freiburg, Germany) for additional 30 min. Pre-treated gels were dried and exposed using a Fujifilm Phosphorimager BAS 1800 II and analyzed with AIDA V3.44.035 densitometry software (Raytest, Straubenhardt, Germany). The band intensity of the samples with (I°_+PK_) and without (I°_-PK_) PK treatment were measured after background subtraction. With respect to samples treated with proteinase K, only bands within the molecular weight range of 18–24 kDa were used for evaluation. The conversion efficiency (CVE) was calculated using the formula CVE [%] = [I°_+PK_°/°(I°_-PK_*10)]*100.

### In vitro aggregation of recombinant PrP

#### Production of recombinant full-length PrP

Recombinant full-length mouse PrP with histidine (wt rPrP) or glycine at positions 60, 68, 76, and 84 (rPrP(TetraH>G)), and 95 (rPrP(H95G)), respectively, was produced and purified essentially as described by Giese *et al*. [42] except that for bacterial expression, BL21(DE3) RIL *Escherichia coli* cells (Novagen) were transformed with plasmids pET17b-PrP23-230 for full-length wt-rPrP, pET17b-TetraH>G-PrP23-230 for TetraH>G-rPrP, pET17b-H95G-PrP23-230 for H95G-rPrP or pET17b-PrP89-230 and pET17b-H95G-PrP89-230 for N-terminal truncated wt-rPrP and H95G-rPrP. Furthermore, for full-length and N-terminal truncated mutant PrPs the nickel-chelate affinity chromatography step was replaced by gel filtration using a Sephadex S-75 HighPrep 16–26 column (GE Healthcare, Munich, Germany) equilibrated with a buffer containing 8 M urea, 10 mM MOPS, pH 7.0, and 150 mM NaCl. Fractions containing purified rPrP were pooled, concentrated with a Centriprep device (Millipore, Schwalbach, Germany) and diluted 1:50 for refolding into 10 mM MES, pH 6.0. Solutions of refolded rPrP were stirred at 4°C for 4–12 h, and aggregates were removed by centrifugation for 30 min at 17,000 rpm at 4°C. Supernatants were concentrated again using Centriprep YM10 ultrafiltration cells (Merck Millipore, Darmstadt, Germany) and cleared from aggregates by centrifugation at 16,000 × g for 30 min at 4°C. Recombinant PrP was finally dialyzed against 2 mM MES, pH 6.0, to remove traces of urea and imidazole. Correctness of the α-helical fold of the purified rPrPs was checked by CD-spectroscopy using a Jasco 715 CD-spectrophotometer. All amino acid numbers refer to the mouse PrP sequence (GenBank^TM^ accession number NP 035300).

#### Fluorescent labelling of rPrP

After buffer-exchange by gel filtration on a microspin column (Mobitec, Göttingen, Germany) filled with Sephadex G15 (Pharmacia Biotech, Sweden) pre-equilibrated with 10 mM sodium phosphate buffer (NaP_i_), pH 6.0, containing 0.1% NP40 (Sigma-Aldrich, Munich, Germany) for two minutes at 700 × g, NaHCO_3_ was added to a final concentration of 100 mM. Protein labelling was performed with amino-reactive fluorescent dyes Alexa Fluor-488-O-succinimidylester and Alexa Fluor-647-O-succinimidylester (Molecular Probes, Invitrogen, Karlsruhe, Germany) were performed exactly as described in Giese *et al*. [42].

#### FCS/SIFT measurements

FCS and SIFT measurements were carried out on an Insight Reader (Evotec-Technologies, Germany) with dual-color excitation at 488 and 633 nm using a 40× of 1.2 NA microscope objective (Olympus, Japan) and a pinhole diameter of 70 μm at FIDA setting exactly as described in Giese et al. [42]. The fluorescence data were analyzed by correlation analysis using the FCSPPEvaluation software version 2.0 (Evotec OAI, Germany). Two color cross-correlation amplitudes G(0) were determined using the same software. Evaluation of SIFT data in two-dimensional intensity distribution histograms was performed as described [42].

#### Aggregation assay

A stock solution of fluorescently-labeled rPrP was prepared in 10 mM NaP_i_, pH 7.2, containing 0.2% sodium dodecyl sulfate (SDS, Sigma) to keep the protein in an unaggregated state at the beginning of the measurements. The concentrations of rPrP-Alexa488 and rPrP-Alexa647 were adjusted to approximately 10 molecules per focal volume and 20 molecules per focal volume, respectively. Experiments were started by diluting this stock solution into NaP_i_-buffer containing 0–0.2% SDS to obtain final SDS concentrations ranging from 0.0125% to 0.2% and a final concentration of labeled rPrP of approximately 5 nM. In some experiments, metal ions (CuCl_2_, MnCl_2_) or EDTA were added to the stock solutions of monomeric rPrP in 0.2% SDS. Experiments were performed in 96-well plates with a cover slide bottom (Evotec Munich, Germany). To reduce evaporation, the 96-well plates were sealed with adhesive film. Typically, aggregation was monitored for 8 h in four parallel identical samples for each experimental group.

### Statistics

Statistical analysis and graphics were done with SigmaStat 2.03, SigmaPlot 6.0 and Microsoft Excel. Data of two independent groups following a normal distribution with equal variances were analyzed with the unpaired Student´s t-test, p ≤ 0.05 was considered statistically significant.

## Results

### Generation and characterization of PrP transgenic mice

**Fig 1:**
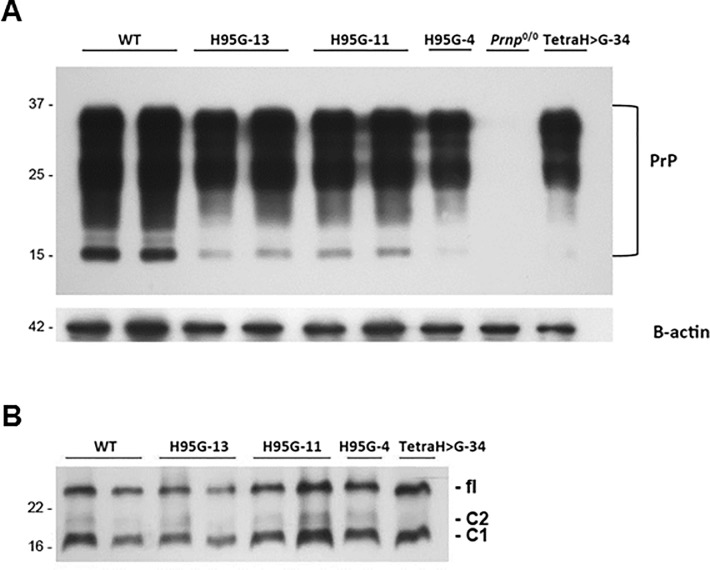
PrP^C^ levels and glycosylation profile in healthy wild-type and transgenic mice expressing mutant PrP. **(A)** Brain homogenates (10 μg per lane) derived from mice expressing full length mouse wild-type (wt), PrP(H95G) lines 4, 11 and 13, PrP(TetraH>G) line 34 and PrP null controls (*Prnp*^0/0^) were subjected to immunoblot analysis using monoclonal antibody SHA31. Blots were reprobed for β-actin to control for equal loading. Molecular weight is indicated on the left (in kDa). (**B**) Removal of N-linked glycans on PrP^C^ encoded by the PrP(H95G), lines 4, 11, and 13 as well as PrP(TetraH>G), line 34 using peptide N-glycosidase F (PNGase F) and probing of the treated samples with monoclonal antibody SHA31. fl = full-length PrP.

We generated transgenic mouse lines encoding full-length mouse PrP with all four histidine residues within the OR region (PrP(TetraH>G)) or at position 95 (PrP(H95G)) replaced with glycine. To this we used the “half genomic” vector, wherein the protein coding sequence of interest is placed under the control of the mouse *Prnp* promoter [5]. Constructs were microinjected into fertilized oocytes on a PrP knockout genetic background (*Prnp*^0/0^, 129/Sv-C57/Bl6), with offspring of four founder lines expanded for further study. PrP(TetraH>G) (line 34) and PrP(H95G)(line 13) mice were examined in detail and found to express the mutant proteins in the brain at a level comparable to full-length PrP^C^ in wt control mice (Fig 1A). Moreover, deglycosylation revealed that PrP^C^ encoded by the PrP(TetraH>G) and PrP(H95G) transgenes was represented by a C1 endoproteolytic fragment as well as full-length protein (*i*.*e*. was processed in a manner analogous to that of wt PrP^C^; Fig 1B). Notably, transgenic mice presented with a wild-type-like distribution of mutant PrP^C^ in the brain, and showed no signs of spontaneous neurodegeneration for an observation period of up to 600 days (exemplarily shown in S1 Fig).

When assessed in transfected RK13 cells (S2A Fig), in sucrose gradient fractionations the TetraH>G version of PrP behaved like PrP^C^ controls and segregated with raft fractions identified by cholera toxin B binding (S2B Fig). Interestingly, however, the TetraH>G allele showed a diminished ability to transduce a toxic signal associated with the accumulation of PrP^Sc^; when SH-SY5Y cells expressing this allele were co-cultivated in the presence of chronically infected N2a neuroblastoma cells they showed a lower tendency to undergo apoptosis than their wt PrP-expressing counterparts (S3 Fig).

### Susceptibility of mice expressing mutant PrP to scrapie prions

To examine the susceptibility of the novel transgenic mice to scrapie, mice descended from PrP(TetraH>G) founder animals (line 34) as well as corresponding wt controls (129/Sv-C57/Bl6) were inoculated intracerebrally (*i*.*c*.) with mouse-adapted Rocky Mountain Laboratory (RML) prion isolate and monitored for the onset of disease-associated symptoms and for survival. Similarly, mice descended from three different PrP(H95G) founders (lines 4, 11, and 13) were also subjected to prion challenge. We found that TgPrP(TetraH>G) mice were susceptible to scrapie, but succumbed to disease significantly later than wt controls (Table 1, "primary passages"). Clinical signs did not appear until 292 days post infection (dpi; line 34), indicating protracted disease duration, but were otherwise similar to those of RML-infected wt mice, with signs including ataxia, kyphosis and difficulties in righting. In contrast, TgPrP(H95G) mice of all three founder lines presented with clinical signs of scrapie, indistinguishable from that of wt control mice, but died after a significantly shortened mean incubation period ranging from 87 to 116 dpi (Table 1, "primary passages"). Notably, PrP^C^ expression in both Tg(TetraH>G) and TgPrP(H95G) lines were comparable to wt levels.

**Table 1:** Response of transgenic mice and corresponding wt controls to mouse scrapie.

Inoculum	Genotype of recipient mice		
	129/Sv-C57/BL6	TgPrP(TetraH>G)	TgPrP(H95G)
**Primary passage**			
RML	150 ± 12 (n = 8)	Line 34: 347 ± 28 (n = 8)	Line 4: 116 ± 3 (n = 8), Line 11: 87 ± 3 (n = 8), Line 13: 89 ± 8 (n = 8)
**Secondary passage**			
TgPrP(TetraH>G)-passaged RML, line HG34 animal #258	160 ± 8 (n = 6)	Line 34: 427 ± 12 (n = 8)	Not determined
TgPrP(TetraH>G)-passaged RML, line HG34 animal #260	153 ± 5 (n = 6)	Line 34: 372 ± 27 (n = 5)	Not determined
TgPrP(H95G)-passaged RML, line 13 animal #304	150 ± 8 (n = 6)	Not determined	Line 13: 75 ± 1 (n = 6)
TgPrP(H95G)-passaged RML, line 13 animal #305	152 ± 4 (n = 6)	Not determined	Line 13: 77 ± 4 (n = 8)

Mean incubation times ± standard deviation in days after *i*.*c*. inoculation (dpi) are presented. For primary transmission mice expressing full-length mouse wt PrP (129/Sv-C57/Bl6) or mutant PrP (PrP(TetraH>G) and PrP(H95G)) were *i*.*c*. inoculated with 30 μl of 10% (wt/vol) RML homogenate in PBS. For secondary passage, brain homogenates from different terminally ill TgPrP(TetraH>G) (line HG34, mouse #258 and 260) as well as TgPrP(H95G) mice (line 13, mouse #304 and 305) were *i*.*c*. inoculated in the same lines of recipient mice.

### Immunohistochemical analyses

**Fig 2:**
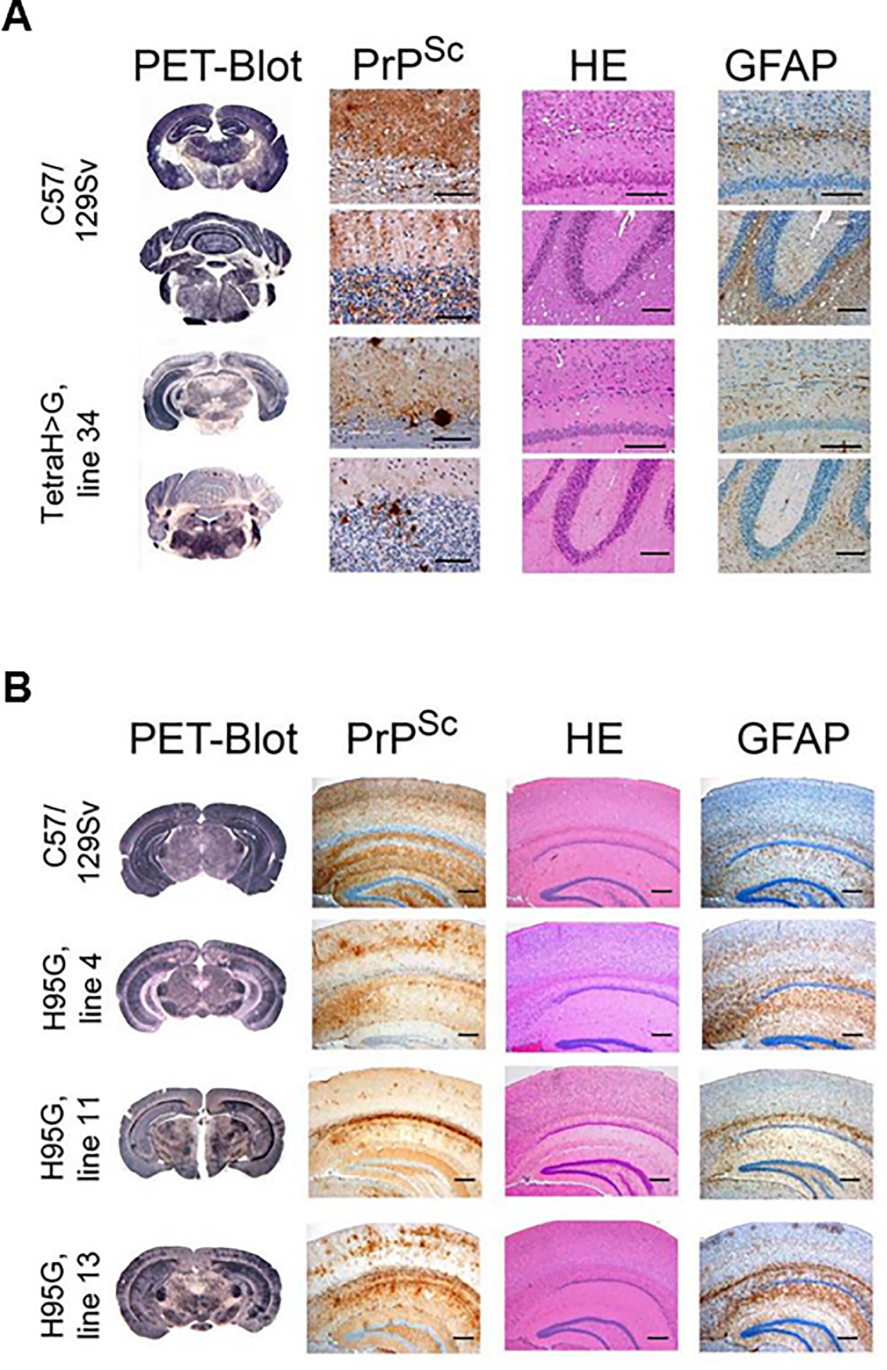
Neuropathological changes and PrP^Sc^ distribution in hippocampal and cerebellar sections of prion-challenged mice. (**A**) First column: Paraffin-embedded tissue (PET) blots demonstrating PrP^Sc^ deposits in hippocampus and cerebellum of RML-infected wild-type (C57/129Sv) and PrP(TetraH>G), line 34 mice. There are plaque-like deposits in and adjacent to the corpus callosum and the internal granular cell layer of the cerebellum in PrP(TetraH>G) mice. Second column: Corresponding PrP^Sc^ immunhistochemistry (upper field: hippocampus and corpus callosum; lower field: cerebellum; monoclonal antibody CDC1; scale bar: 50 μm). Third column: Hematoxylin and eosin stainings (H&E) of hippocampal/corpus callosum and cerebellar sections demonstrating spongiform changes (scale bars of hippocampus/corpus callosum: 100 μm; of cerebellum: 200 μm). Fourth column: GFAP immunostaining demonstrating gliosis (scale bars as in third column). (**B**) Corresponding histopathological and immunohistochemical examination of hippocampal brain sections derived from terminally ill PrPH95G mice and corresponding wt controls (scale bars: 200 μm). Three independent Tg lines (lines 4, 11 and 13) were assessed for these analyses.

Brain sections from terminally ill RML-infected transgenic and wt control mice were examined for pathological changes. Wild-type mice (n = 6) presented with spongiform changes that were prominent in the hippocampus, thalamus, septum, cortex and throughout all white matter areas (Fig 2A, upper two rows). This was accompanied by distinct synaptic PrP^Sc^ deposits. Diseased TgPrP(TetraH>G) mice demonstrated a wild-type-like distribution of spongiform changes with considerably less severe spongiosis (Fig 2A, lower two rows, third column). Concordantly, there was a tendency to milder gliosis in some mice (Fig 2A, lower two rows, fourth column). Immunostaining, however, revealed a PrP^Sc^ deposition pattern in TgPrP(TetraH>G) mice markedly different from the predominantly synaptic pattern seen in wt controls. Instead, plaque-like accumulations of PrP^Sc^ with a dense globular core were observed throughout all sections, particularly noticeable adjacent to the corpus callosum (Fig 2A, lower two rows, first and second columns).

Regarding terminally ill TgPrP(H95G) mice, spongiform changes were significantly less pronounced in all grey and white matter areas analyzed. In all three TgPrP(H95G) transgenic lines, the most extensive spongiform changes were found in the hippocampus and thalamus, while cortex and white matter areas were relatively spared (Fig 2B, rows 2–4, third column). Compared to RML-infected wt mice (Fig 2B, upper row) the thalamus, hippocampus and septum of TgPrP(H95G) mice were similarly affected by gliosis, whereas the medulla and the thalamic cortex tended to higher or lower scoring values, respectively (Fig 2B, rows 2–4, third column). Again, striking observations applied to the staining for PrP^Sc^ deposits, which in TgPrP(H95G) mice appeared as feathery plaque-like structures on a faint synaptic background (Fig 2B, rows 2–4, second column), with accumulations also revealed by the PET-blot technique (Fig 2B, rows 2–4, first column). The most prominent pathology was recognized in TgPrP(H95G) mouse line 13, followed by lines 4 and 11. Prior studies have indicated that clinical and pathological manfestations of experimental scrapie disease in mice are little affected by chronological age of the host [43, 44], indicating that these different patterns of onset and pathology more likely relate to intrinsic properties of the agent than age-dependent host responses.

### Lesion profiling

**Fig 3:**
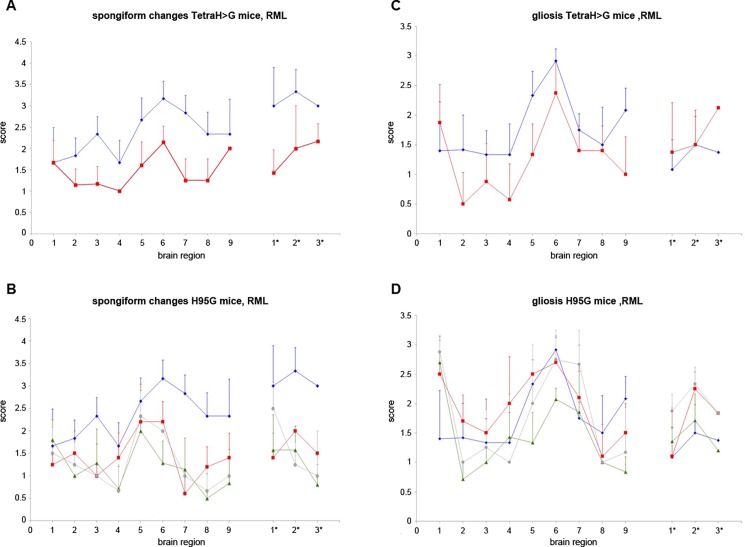
Lesion profiles induced by mouse-adapted scrapie isolate RML in wild-type and transgenic mice expressing mutant PrP. The extent of spongiform change (**A**) and reactive gliosis (**C**) in brain sections of terminally ill wt and PrP(TetraH>G) was assessed semi-quantitatively in a blinded fashion in nine areas of grey matter and three areas of white matter by lesion profiling. Animals were scored on a scale of 0–5 in each specific area, and mean scores (n = 6 (C57/129Sv), versus n = 7 (PrP(TetraH>G), line 34), respectively) are shown graphically (error bars plus SD). Blue diamonds: C57/129Sv. Red squares: PrP(TetraH>G). Analogous data from PrP(H95G) mice are shown in panels **B** and **D** (n = 4 (PrP(H95G), line 13), n = 5 (PrP(H95G), line 4) and n = 7 (PrP(H95G), line 11), respectively); data for C57/129Sv wt animals has been re-plotted for comparative purposes. Blue diamonds: C57/129Sv. Red squares: PrP(H95G), line 4. Green triangles: PrP(H95G), line 11. Grey circles: PrP(H95G), line 13. Scoring areas as follows: Grey matter: 1, dorsal medulla, 2, cerebellar cortex, 3, superior colliculus, 4, hypothalamus, 5, medial thalamus, 6, hippocampus, 7, septum, 8, medial cerebral cortex at septum level, 9, medial cerebral cortex at thalamus level. White matter: 1*, cerebellar white matter, 2*, mesencephalic tegmentum, 3* pyramidal tract.

Using established protocols, we scored the extent of spongiosis and gliosis within 12 brain regions of the infected mice in a blinded fashion. These analyses, to assess allelic effects upon neuropathological endpoints, are shown in Fig 3. With the exception of region 1 (dorsal medulla), the lesion profiles of spongiform change and gliosis in PrP(TetraH>G) mice occurring after *i*.*c*. inoculation of RML were similar in shape but with smaller magnitude than in non-Tg mice (Fig 3A and 3C). For the H95G allele, the overall profile of lesions resembled wt mice of the C57/129 genetic background but, again, the intensity of the lesions was attenuated (Fig 3B); gliosis was similar to wt (Fig 3D).

### Immunodetection of PrP^Sc^

**Fig 4:**
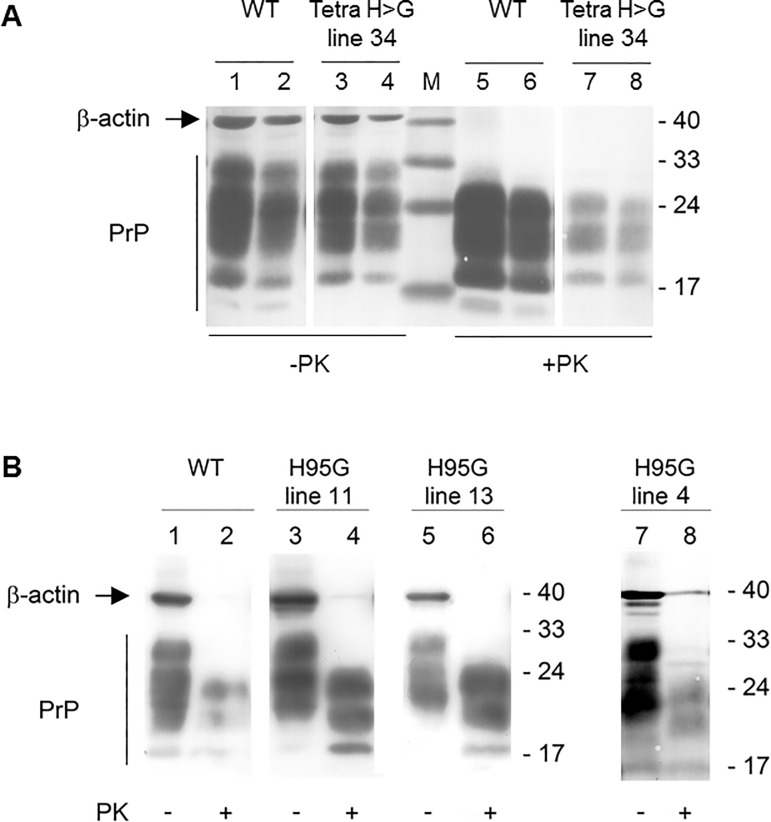
Western blot analysis of brain lysates from RML-infected wt and transgenic mice for total PrP^C^ and PrP^Sc^ using monoclonal antibody 4H11. (**A**) Brain homogenates from terminally ill wt and PrP(TetraH>G) line 34 mice were either left untreated (- PK) or subjected to digestion with proteinase K (+ PK). Blots were reprobed for β-actin to control for equal loading. Bands corresponding to total PrP are marked on the left. Irrelevant lanes have been excised at two positions. Molecular weight standards are given on the right (in kDa). (**B**) Corresponding immunoblot analysis of brain homogenates extracted from RML-infected PrP(H95G) mice from the three different lines 11, 13, and 4, respectively, and corresponding wt control (lanes 1 and 2) before (-) and after (+) treatment with PK. Molecular weight standards are given on the right (in kDa).

Western blot analysis of brain tissue derived from terminally ill transgenic mice revealed that all animals harboured protease-resistant PrP^Sc^ in their brains (Fig 4). TgPrP(TetraH>G) line 34 mice propagated PrP^Sc^ with similar ratios of putative di-, mono- and unglycosylated forms to those seen in RML-infected wt mice (Fig 4A). This was also the case for TgPrP(H95G) mice descended from three different founder animals (Fig 4B). In terms of quantity, the amount of PrP^Sc^ was notably diminished versus wt for TgPrP(TetraH>G) line 34.

### Properties of prions after sequential passage in TgPrP(TetraH>G) and TgPrP(H95G) mice

We next investigated if sequential passages of a prion isolate in Tg mice resulted in the emergence of novel biological properties. Brain tissues derived from PrP(TetraH>G) and PrP(H95G) mice that were scored as positive for prion infection (by typical clinical signs, abnormal PrP immunohistochemistry, and the presence of PrP^Sc^ on western blot analysis) were used to *i*.*c*. inoculate recipient mice of the same genotype. In one set of analyses, PrP(TetraH>G) (line 34) and wt mice were inoculated with brain homogenate extracted from two RML-infected PrP(TetraH>G) line 34 mice. In TgPrP(TetraH>G) mice challenged with PrP(TetraH>G)-passaged prions, the prolonged incubation times observed with the primary transmission of RML prions in corresponding host mice were again apparent, exceeding 350 days (Table 1, "secondary passages"). In contrast, wt mice challenged with PrP(TetraH>G)-passaged prions presented with survival times that were not significantly different from the corresponding RML-inoculated group (p = 0.08 for inoculum #258 and p = 0.47 for inoculum #260); see Table 1, "secondary passages"), indicating no loss in infectious titre.

**Fig 5:**
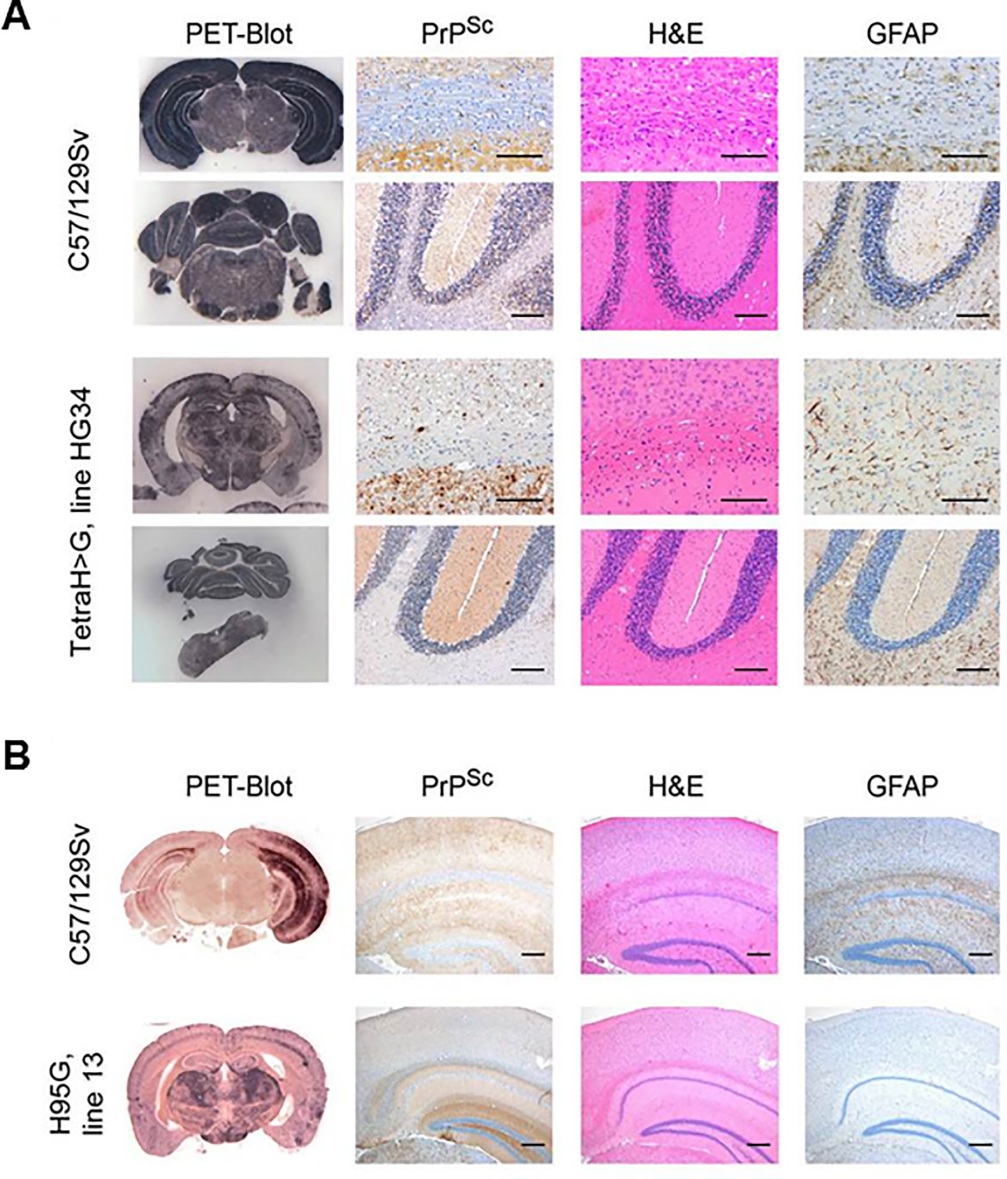
Neuropathological changes and PrP^Sc^ distribution pattern in brain sections of secondary passage transgenic mice and corresponding wt controls. (**A**) First column: Paraffin-embedded tissue (PET) blots demonstrating PrP^Sc^ deposits in hippocampus (upper panels) and cerebellum (lower panels) of wt (C57/129Sv) and PrP(TetraH>G), line 34 inoculated with PrP(TetraH>G)-passaged prions. Second column: Corresponding PrP^Sc^ immunhistochemistry (mAb CDC-1). Third column: Hematoxylin and eosin stainings (H&E) of hippocampal and cerebellar sections demonstrating spongiform changes. Fourth column: GFAP immunostaining demonstrating gliosis. (**B**) Corresponding hippocampal brain sections from terminally ill PrP(H95G) mice (line 13) and corresponding wt controls (C57/129Sv) challenged with PrP(H95G)-passaged prions.

PrP(H95G) (line 13) and wt animals were infected with brain homogenate derived from two terminally ill line 13 PrP(H95G) mice. In this secondary passage experiment, a non-significant trend towards reduced survival times was observed in PrP(H95G) mice compared to the initial RML-infected group (Table 1, "secondary passages") however, no significant change in survival time could be observed with wt mice inoculated with PrP(H95G)-passaged prions compared to the corresponding RML-infected control group (Table 1, "secondary passages"). Neuropathological lesions and the pattern of PrP^Sc^ deposition in the brains of secondary passage transgenic mice and wt control animals resembled those noted for the corresponding RML-infected groups (Fig 5). Moreover, western blot analyses of brain tissue derived from transgenic mice expressing mutant PrP as well as wt mice inoculated with PrP(Tetra-H>G)- and PrP(H95G)-passaged prions, revealed a banding pattern corresponding to total PrP and PrP^Sc^, respectively, which resembled the initially RML-infected groups (not shown).

### *In vitro* conversion capability of full-length PrP(TetraH>G)

**Fig 6:**
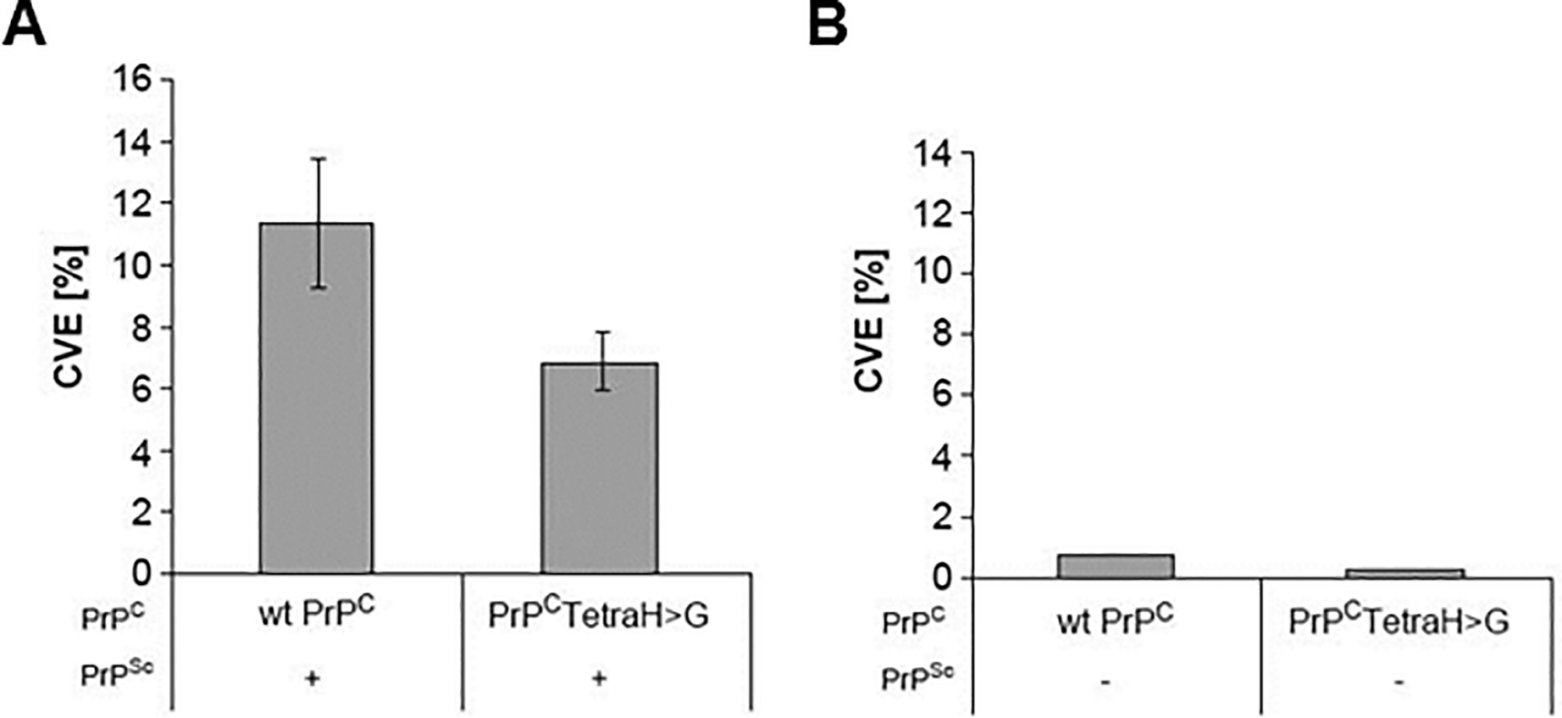
Conformational changes measured by *in vitro* conversion reactions. **(A)** *In vitro* conversion reactions have been performed with radiolabeled wild-type and PrP^C^(TetraH>G) purified from RK13 cells and PrP^Sc^ purified from brains of RML-infected Tga20 mice as described [4]. Samples were analyzed by SDS-PAGE-fluorography, and relative conversion efficiencies (CVE) were calculated from band intensities before and after digestion with proteinase K using the formula CVE [%] = [I°_+PK_ / (I°_-PK_*10)]*100. PrP^C^ with substituted OR histidines (PrP^C^(TetraH>G)) is only half as efficient in converting to the misfolded, PK-resistant conformer than wt PrP^C^. Mean values ± standard error (SEM) were determined from 11 independent experiments for each PrP^C^ type. P-values (p (two sided) = 0.07, p (one sided) = 0.036) were obtained by T-Test calculation. (**B**) Control reactions performed in the absence of PrP^Sc^ seed.

To assess whether extended incubation times in Tg PrP(Tetra-H>G) mice represented a compromised ability of the mutant PrP^C^ to convert to the PK-resistant conformation (“PrP^res^”), we performed *in vitro* reactions in the presence of purified brain-derived PrP^Sc^ using the method of Kocisko *et al*. [4]. In this approach, the altered PrP^C^ variant as well as wild-type PrP^C^ from mouse were expressed in PrP^C^-deficient cells in the presence of ^35^S-methionine and ^35^S-cysteine, purified by immunoprecipitation, and used together with purified PrP^Sc^ from RML infected mouse brain for *in vitro* conversion reactions. We found that substitution of all four OR histidines reduced the conversion efficiency obtained with wt PrP almost two-fold (Fig 6A; p = 0.07 and 0.03 using two-sided and one-sided T-test analyses, respectively). There was no detectable PrP^res^ in unseeded reactions (Fig 6B), indicating that neither mouse wt PrP nor PrP(TetraH>G) developed intrinsic resistance towards proteinase K digestion under these assay conditions.

### Single molecule analysis of glycine-substituted PrP alleles

In a final set of analyses, we assessed the ability of single molecules of recombinant full-length wt PrP and the two mutant proteins to undergo structural conversion and aggregate formation in the presence of putative co-factors [45, 46]. To quantify and characterize rPrP aggregates formed, we used fluorescently-labelled PrP and cross-correlation analysis as well as scanning for intensely fluorescent targets in a confocal single molecule detection system, as described previously for recombinant full-length human PrP [42]. The use of single particle fluorescence in combination with a measurement setup optimized for high-throughput measurements in multi-well plates allowed a large number of individual measurements to be performed, a potentially advantageous approach given inherent variability often observed in assays of *de novo* protein aggregation.

**Fig 7:**
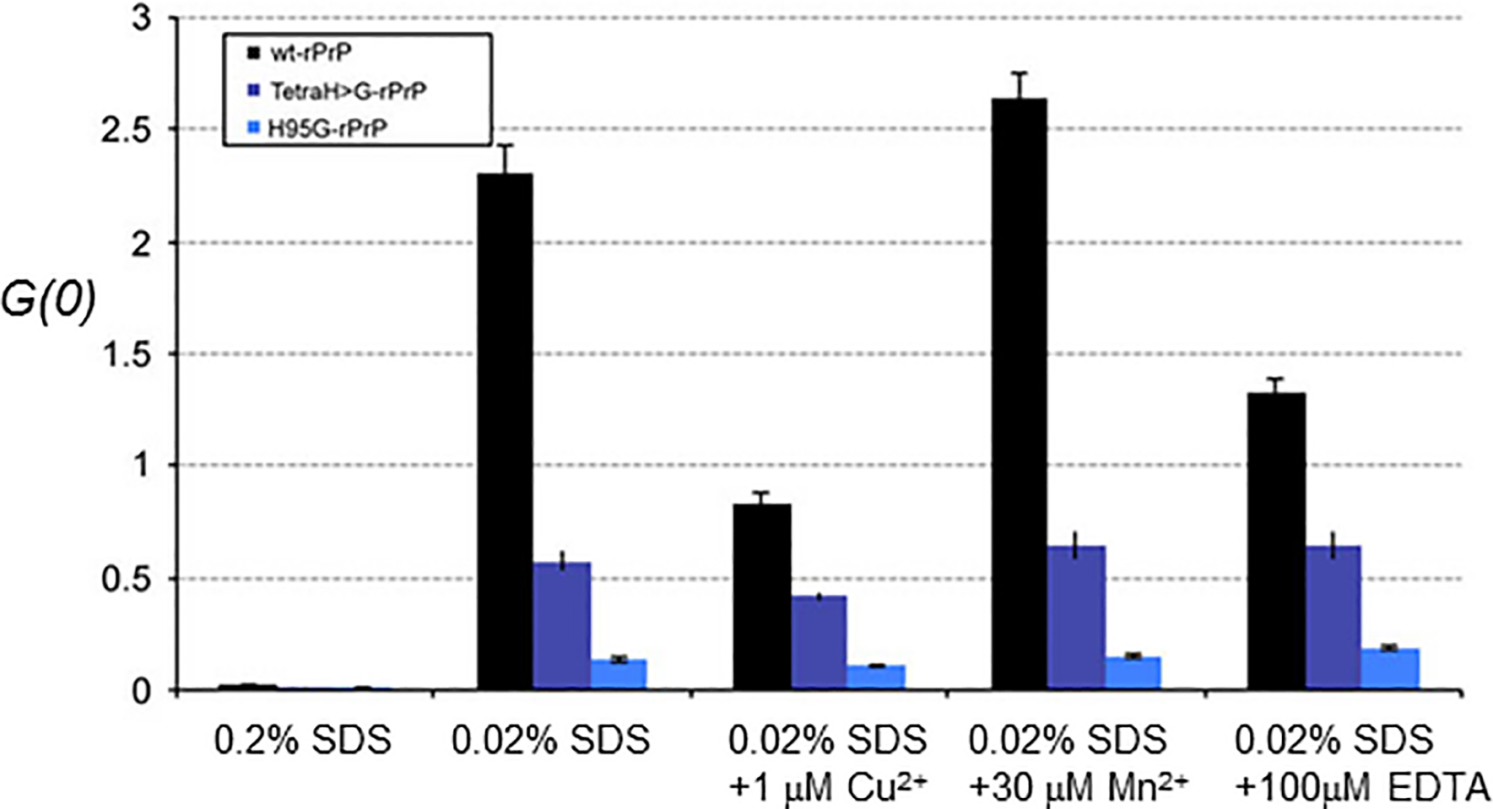
Conformational changes measured by fluorescence correlation spectrometry (FCS). The y-axis represents the *G(0)*, two-color cross-correlation amplitude values. *In vitro* aggregation of recombinant full-length mouse wild-type or mutant PrP analyzed by FCS. Wild-type (wt-rPrP, black columns) and mutant rPrP (TetraH>G-rPrP, purple columns, and H95G-rPrP, blue columns) showed no aggregation at 0.2% SDS. Decreasing the SDS concentration to 0.02% resulted in aggregation of all recombinant proteins to a different extent, being highest with wt-rPrP and lowest with H95G-rPrP. Addition of metal ions like copper (Cu^2+^) and manganese (Mn^2+^) as well as depletion of divalent metal ions with EDTA in the presence of 0.02% SDS had a much stronger impact on the aggregation of wt-rPrP than on TetraH>G-rPrP and H95G-rPrP. Data are shown as mean values of last five meanders ± SEM of four to six independent experiments with four replicates each.

Wild-type rPrP and the two mutant proteins (i.*e*. TetraH>G-rPrP and H95G-rPrP) were all found in the non-aggregated state in 0.2% SDS at pH 7.2 in the absence of additional copper (S4 Fig, "0.2% SDS"), the results for the wt allele being anticipated from prior studies [45, 46]. After SDS was diluted out below 0.05% to induce aggregate formation, an increase in the cross-correlation amplitude was observed, which is a direct measure for the presence of double-labeled particles present in the sample, and is thus indicative of *de novo* oligomerization. Thus, at SDS-concentrations of 0.02%, wt rPrP reached a significant higher cross correlation amplitude than TetraH>G-rPrP and in particular H95G-rPrP (Fig 7, "0.02% SDS"), indicating that the mutations reduced the oligomerization propensity of wt PrP.

Disturbances in the levels of copper and manganese have been described in prion-infected brain tissue [47–49] and changes in brain copper levels have been suggested to influence incubation time in experimental prion disease [11, 13]. To assess whether metal ions such as Cu^2+^ and Mn^2+^ affect the aggregation properties of wt and mutant rPrP, we added different amounts of CuCl_2_, MnCl_2_, and EDTA to aggregation reactions in the presence of the 0.02% concentration of SDS that is permissive to formation of PrP multimers. As shown in Fig 7, in the presence of 0.02% SDS, copper concentrations of 1 μM (in molar excess to recombinant protein) repressed the aggregation of wt-rPrP about 70%, whereas the aggregation of both TetraH>G-rPrP and H95G-rPrP was less affected (Fig 7, "1 μM Cu^2+^"). Higher copper concentrations (10 μM and 50 μM) were able to further decrease the aggregation efficiency of wt-rPrP but again showed no significant effect when added to mutant rPrPs (data not shown). These data indicate that sequences within and adjacent to the OR region in unmetallated PrP (i.e., apo-PrP) facilitate aggregation, but this effect is offset by metallation. TetraH>G-rPrP and H95G-rPrP assessed under the same conditions have lower baseline measures for aggregation that are little affected by Cu^2+^ addition. To assess whether a reported stimulatory effect of Mn^2+^ on aggregation of human rPrP(23–231) [42] is also applicable for full-length mouse rPrP, we added MnCl_2_ in the same type of protein aggregation experiments. We found that manganese concentrations of 30 μM were able to further increase the aggregation of full-length wt rPrP by ~15%, with a mild proportional increase noted for the TetraH>G-rPrP allele but not the H95G-rPrP allele (Fig 7, "30 μM Mn^2+^"). To assess whether the buffer used for aggregation assays contained traces of divalent metal ions that affect outcome measures, we performed controls using the chelating agent EDTA. In the presence of 100 μM EDTA wt-rPrP aggregated ~40% less than with 0.02% SDS alone (Fig 7, columns labelled “100 μM EDTA”). Addition of the chelating agent did not completely abolish the inhibitory effect seen with Cu^2+^, nor the stimulating effect seen with Mn^2+^. Again, no affect was seen on the aggregation of the mutant proteins.

Taken together, our data indicate that H>G replacements in the OR region and at position 95 resulted in a decreased tendency for aggregation of full-length mutant proteins versus the wt point of reference.

## Discussion

### Multiple activites of the PrP^C^ OR

Prior work has defined complex relationships between the PrP^C^ OR region and prion disease pathogenesis. Though the OR region comprises the main Cu-binding region of PrP^C^, accommodating up to 4 Cu (II) ions per polypeptide chain, a direct causative link between copper and prion disease was challenged by the observation that transgenic mice completely lacking the OR region (PrPΔ32–93) were still susceptible to infection with laboratory prion isolates [6]. Elimination of the OR region slowed the rate of disease progression in these transgenic mice, indicating that the octarepeats are not necessary for sustaining prion replication and pathogenesis, but do modulate the extent of neuropathological degeneration and disease presentation. Genetic prion diseases are associated with extra octarepeats (*i*.*e*., above the typical wt complement of one histidine-lacking OR followed by four histidine-containing ORs), with these mutant PrP alleles likely originating from replication fork slippage on tandem direct repeats during DNA replication. For these open reading frame expansions, the more octapeptide repeats are present in PrP, the more prone the protein is to misfolding [50, 51], which correlates with earlier disease onset and shorter disease duration in affected patients [52]. Also, when supernumerary octarepeat insertions rise above a threshold, they give rise to GSS-like disease with cerebral amyloid deposition instead of the clinical picture of familial CJD [53]. This effect, seen with histidine-containing wt OR units, has been ascribed to a conformational aspect of metal-binding to the OR region, where insertions impede the ability to transition from a configuration denoted "component 3" to a multivalent configuration, "component 1" [54]. Overall, an extensive literature contains complex, and sometimes contrasting, findings [55–58]; taken together one may surmise that the OR region has pleiotropic effects, *i*.*e*. modulating prion replication and pathogenicity, as well as producing a protective effect against oxidative stress [59, 60] and modulating copper-dependent multimerization [61]. To address these complex relationships and the *in vivo* role of site 5 we created two new strains of transgenic mice. The resulting Tg lines revealed a number of unexpected features as well as some parallels to mice with structure-guided re-design of the PrP OR region [26].

### Biological properties of PrP(TetraH>G)

We made a line of PrP(TetraH>G) mice that expressed PrP^C^ at a level slightly less than that seen in wt control animals. Here, the histidine to glycine substitutions within the OR region did not influence α-cleavage in the brain to make C1 fragment (Fig 1); others have noted that copper-stimulated PrP internalization requires both the octapeptide repeat domain and the palindromic sequence starting at the α-cleavage site (amino acids 110/111 in mouse PrP) to be intact [18, 23, 62]. In terms of cell biological effects, PrP(TetraH>G) had a diminished capacity to initiate cell death when exposed to an environment containing PrP^Sc^ and under cell culture conditions without copper-supplementation, PrP(TetraH>G) trafficked to lipid rafts like wt PrP^C^ (S2 and S3 Figs). While we cannot exclude that Cu-dependent endocytosis would be impaired for this allele, this process has been modeled in other studies by supplementation of culture medium with copper concentrations of 100–500 μM, considerably higher than for the other studies described here [23, 63].

For the biology of metal speciation, Miura *et al*. have suggested that a possible role of the wt octarepeat motif is to stabilize the α-helical structure of PrP^C^ in the presence of copper [64]. Also, using hybrid density functional theory (DFT) calculations, Hodak *et al*. have inferred that at physiologically-relevant low Cu(II) concentrations, a structural alteration of the Cu-bonded octarepeat region creates flexible turns in the areas separating copper-bound residues, which increases the rigidity of the entire N-terminal domain to make it more resistant to misfolding [12]. On the other hand, and bearing in mind that mature wt PrP^C^ is normally considered to exist a monomer, Yen *et al*. reported that copper-induced structural conversion of wt PrP^C^ predisposes it for oligomerization that is associated with toxicity [58]. Thus, we arrive at opposite predictions that in the presence of copper, the OR region of the PrP(TetraH>G) allele is now susceptible to misfolding under low Cu(II) occupancy or is prevented from Cu-dependent oligomerization. In practice, and making an assumption that PrP^C^ can become charged with endogenous bioavailable metal [65], we could not detect a spontaneous neurodegenerative disease in the aged PrP(TetraH>G) Tg mice (not shown). In the case of prion-infection initiated with *i*.*c*. delivery of PrP^Sc^—a situation where PrP^C^ does misfold and multimerize—replacement of the four wt histidine residues within the OR region with glycine residues resulted in prolonged survival times of scrapie-infected PrP(TetraH>G) mice (Table 1). These results fit well to the finding that treatment of mice with the copper chelator D-penicillamine (D-PEN) delays onset of prion disease [13]. In addition, the diseased animals show reduced PrP^Sc^ levels and serial passage of infectivity from the brains of mice with end-stage disease resulted in prolonged incubation times in comparison to the parental stock of RML prions, indicative of a drop in infectious titre. Our overall results with PrP(TetraH>G) line 34 mice indicate that the amino acid exchange not only influenced the OR itself but also induced a persistent conformational change in the C-terminal protease-resistant core portion of the protein. These results can be reconciled with our *in vitro* data showing that recombinant PrP(TetraH>G) was less susceptible to SDS-induced aggregation than wt rPrP, and that cellular PrP(TetraH>G) had a reduced propensity for conformational *in vitro* conversion to the protease resistant isoform (Figs 6 and 7, S4 Fig). Perhaps most remarkably, we found plaque-like PrP^Sc^ deposits with a dense globular core in infected TgPrP(TetraH>G) mice. A second PrP(TetraH>G) Tg line designated HG1-2CL was profiled less extensively but behaved in a similar manner to line 34 in the accumulation of plaque deposits (as well as protease-resistant PrP profile after RML inoculation and in the PMCA conversion assay; not shown). As the dimorphism L108F, T189V that defines the mouse *Prnp*^b^ allele is typically required to allow certain prion isolates to make amyloid plaques in mice [44, 66, 67], this phenomenon is most unusual for prion infections where the PrP^C^ precursor has C-terminal sequences identical to the mouse *Prnp*^a^ allele.

### Biological properties of PrP(H95G)

In contrast to the prolonged incubation times observed with scrapie-infected PrP(TetraH>G) mice compared to wt mice, TgPrP(H95G) mice had more rapid disease progression. Cox *et al*. have suggested from *ab initio* electronic structure calculations of rPrP(89–230) that detachment of copper bound to H95 might be a pivotal step in the conversion pathway [68]. According to this model, ablation of copper binding to H95 with Ala, Tyr or Gly substitutions should present with shortened incubation times; for the H95G allele here, this was indeed the case (Table 1). *In vitro* analyses of site 5 mutations using extended X-ray absorption fine structure spectroscopy (EXAFS) along with studies in cultured cells provided some overlapping findings and conclusions [69]. These authors showed enhanced PK-resistance of an epitope-tagged mouse PrP equivalent to H95Y when expressed in chronically infected neuroblastoma cells and also that this allele expressed in uninfected cells generated misfolded PrP that could "seed" uninfected recipient N2a cells. These studies proposed a mechanism underlying these effects wherein a pH drop caused by trafficking into endosomal/lysosomal compartments causes H95Y PrP to "switch" to a pathological conformation with enriched β-sheet content in the segment spanning residues 105–116 and the adjacent palindromic motif. Conversely, WT PrP with the non-OR site 5 region coordinated in the 2His state (i.e. copper coordinated by His95 and His110) is stabilized by an attenuation of contacts between residues 89–126 and the C-terminal structured domain. Furthermore, according to the computational model in Cox *et al*., copper coordination to H95 can protect PrP from prion conversion as in their copper binding at this position is not compatible with formation of β-strands in a model for PrP^Sc^ involving left-handed helices [68]. However, not exactly in accord with these *in silico* and *in vitro* analyses [68, 69], the shortened disease progression noted in TgPrP(H95G) mice could neither be ascribed to an increased tendency to undergo structural conversion and aggregate formation triggered by a reduction of SDS concentration from 0.2 to 0.02% in an *in vitro* system, nor to increased PrP^Sc^ levels in the brains of terminally ill mice (assessed by western and PET blot analyses, respectively). Given that PrP^Sc^ itself is not necessarily the primary cause of neurodegeneration and that aggregation, toxicity and infectivity are distinct processes (summarized in [70]), it appears either that PrP(H95G) aggregates are more toxic than aggregated wt (or for that matter PrP(TetraH>G)) or that copper depletion at position 95 may play a role in conferring PrP^Sc^ neurotoxicity. In accord with the concept of altered toxic behaviour, diffuse PrP deposition observed in TgPrP(H95G) mice differed from the synaptic pattern seen in scrapie infected wt mice and, in contrast to TgPrP(TetraH>G) mice, more closely resembling genetic human prion diseases arising from point mutations in the C-terminal domain [71].

### Relationships between PrP N-terminal function and disease pathogenesis

Although studies here and in a related paper [26] were undertaken to explore different predictions from *in vitro* studies, overlaps were seen in outcomes measured in the brains of Tg mice. The PrP(S3) allele made by Lau et al. has changes (within each OR of the OR region) that will accentuate and lock "component 3" binding geometry [26]; this geometry is associated with nM Cu-binding affinity and a metal: polypeptide stoichiometry of 1:1. A PrP S1 allele made by Lau et al. has a different OR region alteration to disfavor this type of copper binding. Here, the PrP(H95G) substitution C-terminal to the OR region was designed to attenuate “site 5” copper binding (also with a metal:polypeptide stoichiometry of 1:1 and which has a *K*_d_ estimated between 10^−9^–10^−14^ M in its wt form [72, 73]). In practice, and accommodating for transgene expression levels, both PrP(S3) and PrP(H95G) alleles led to curtailed incubation periods upon prion inoculation, with a focus upon altered toxicity rather than elevated infectious titre; indeed, serial passage of infectivity and lower spot counts in a scrapie cell assay indicated depressed infectious titres for PrP(S3) [26] while inocula from TgPrP(H95G) mice produced typical incubation times in wt mice (Table 1). For PrP(TetraH>G) and PrP(S1), these alleles were designed to alter the OR region in different ways: in the case of PrP(TetraH>G), to remove OR histidines and prevent any metallation (and hence any metal-dependent structural compaction) of the OR region while in the case of PrP(S1), to hold all histidine-containing OR's in an extended conformation [25, 26]. Here, in practice, and again accommodating for transgene expression level, both alleles were associated with reduced PrP^Sc^ levels, reduced titre and prolonged incubation times subsequent to prion inoculation.

One school of thought is that distinct redox activites of PrP are involved in the mechanisms underlying prion diseases by modulating reactions leading to ROS-mediated β-cleavage and initiating beta-sheet formation. However, the finding that cleavage in the vicinity of the β site can be greatly enhanced by a process independent of metal binding to the OR region [26, 74] gives pause to the view that redox processes are necessarily front and centre in PrP biology. Instead, conformational changes may lie to the fore. While the OR region and site 5 are natively unstructured in apo-PrP, we conclude that *cis* interactions between the OR region or the region corresponding to site 5 and the globular C-terminal domain can modulate pathogenic outcomes. This conclusion is based in part upon *in vitro* studies where the OR region in zinc-loaded PrP interacts with negatively charged residues on helices 2 and 3 [75] and where Cu-loaded OR region (in component 3 geometry and with site 5 inactivated by histidines to tyrosine replacements) interacts with helix 3 [76]. Using a different approach, Thakur *et al*. inferred that in the presence of copper the PrP OR region interacts with helix 2 and the site 5 region interacts in *cis* with helix 1 and the preceeding loop [77]. For the folding of PrP expressed in the brain, the PrP^Sc^ from TgPrP(S3) mice with an altered OR had a change in accessibility of the 12B2 epitope at residues 89–93 closely adjacent to H95 [26] and *cis* effects upon the globular domain could be inferred from altered infectivity titres, proteinase K-resistance and responses in conformational stability assays. We infer that similar long-range remodeling effects likely drive the outcome measures in Tg.PrP(TetraH>G) and Tg.PrP(H95G) mice. Moreover, since the vicinity of site 5 is also a major binding site for PrP^C^/oligomeric Aβ interactions [78, 79] further work to understand these interactions may be useful above and beyond the immediate context of prion infections.

## Supporting information

S1 Fig Wt-like properties of the PrPTetraH>G allele assessed in Tg mice. TetraH>G line 34 mice did not develop cerebellar lesions or dysmorphology that distinguished them from non-Tg control wt C57/129Sv mice.
(TIF)

S2 Fig Cellular localization and expression level of full-length mouse wt PrP^C^ or TetraH>G PrP expressed in RK13 cells. (**A**) Expression level and glycosylation pattern of wild-type and mutant PrP in RK13 (left panel) respectively, monitored by Western blot analysis using monoclonal antibody 6H4 after SDS-PAGE of whole cell lysates before (-) and after (+) treatment with PNGase F. Molecular weights are indicated on the left (in kDa). (**B**) Localization of wild-type and mutant PrP to lipid rafts. RK13 cells transfected to express full-length mouse PrP (left panel) or PrP(TetraH>G) (right panel) were cultivated to confluence and then placed on ice or kept at 37°C. Sucrose gradient flotation was done with cell lysates. Aliquots of each fraction collected from the top of the gradient were precipitated, subjected to SDS-PAGE and immunoblot analysis using antibody RA3153. Dot Blot analysis with horseradish-peroxidase conjugated cholera toxin subunit B (Molecular Probes®, Leiden, Netherlands) was performed to detect the lipid rafts containing fractions.
(TIF)

S3 Fig Co-cultivation assay for determining toxic effects of PrP^Sc^. (**A**) SH-SY5Y cells expressing full-length wild-type PrP (PrP), or PrP(TetraH>G), PrP(H95G) or GPI-coupled GFP (GPI-GFP) were co-cultured with prion-infected (ScN2a) or uninfected neuroblastoma cells (N2a) for 16 h. For quantification of apoptotic cell death, SH-SY5Y cells were fixed, permeabilized and stained for active caspase-3. The percentage of apoptotic cells among transfected cells is shown. (**B**) Expression of transfected constructs analyzed by Western blotting using the anti-PrP antibody 4H11 or an anti-GFP antibody. Molecular weights (kDa) are given on the left. *** p < 0.0005.
(TIF)

S4 Fig *In vitro* conversion of PrP with disrupted copper binding sites within the OR region using protein misfolding cyclic amplification (PMCA). Prior to PMCA, healthy brain homogenates were adjusted to 60 ng PrP^C^/μl with 10% (wt/vol) PrP^0/0^ brain homogenate, spiked 1:50 with 10% (wt/vol) RML homogenate and subjected to 10 rounds of PMCA. *In vitro* generated wt PrPres (C57/129Sv; lanes 1–4), PrPres(TetraH>G) lines 34 (lanes 5–8), as well as PrPΔ32–93 (C4; lanes 9–12) was detected using monoclonal antibody 4H11. In order to rule out degradation of PrP^C^ before PMCA, the corresponding undigested starting material was loaded as control (lane 13: wt; lane 14: PrP(Tetra H>G), line 34, lane 15: C4). Molecular weights are indicated on the right (in kDa). All samples were blotted onto the same membrane and exposed for the same amount of time; irrelevant lanes have been excised at two positions.
(TIF)

S1 Text Supporting material and methods. (DOC)

S2 Text Supporting results. (DOC)

S3 Text Supporting references. (DOC)
