# Supplementary figures and images for: Substitutions of PrP N-terminal histidine residues modulate scrapie disease pathogenesis and incubation time in transgenic mice
Source: PLoS One. 2017 Dec 8;12(12):e0188989. doi: 10.1371/journal.pone.0188989 (PMC5722314; doi:10.1371/journal.pone.0188989)

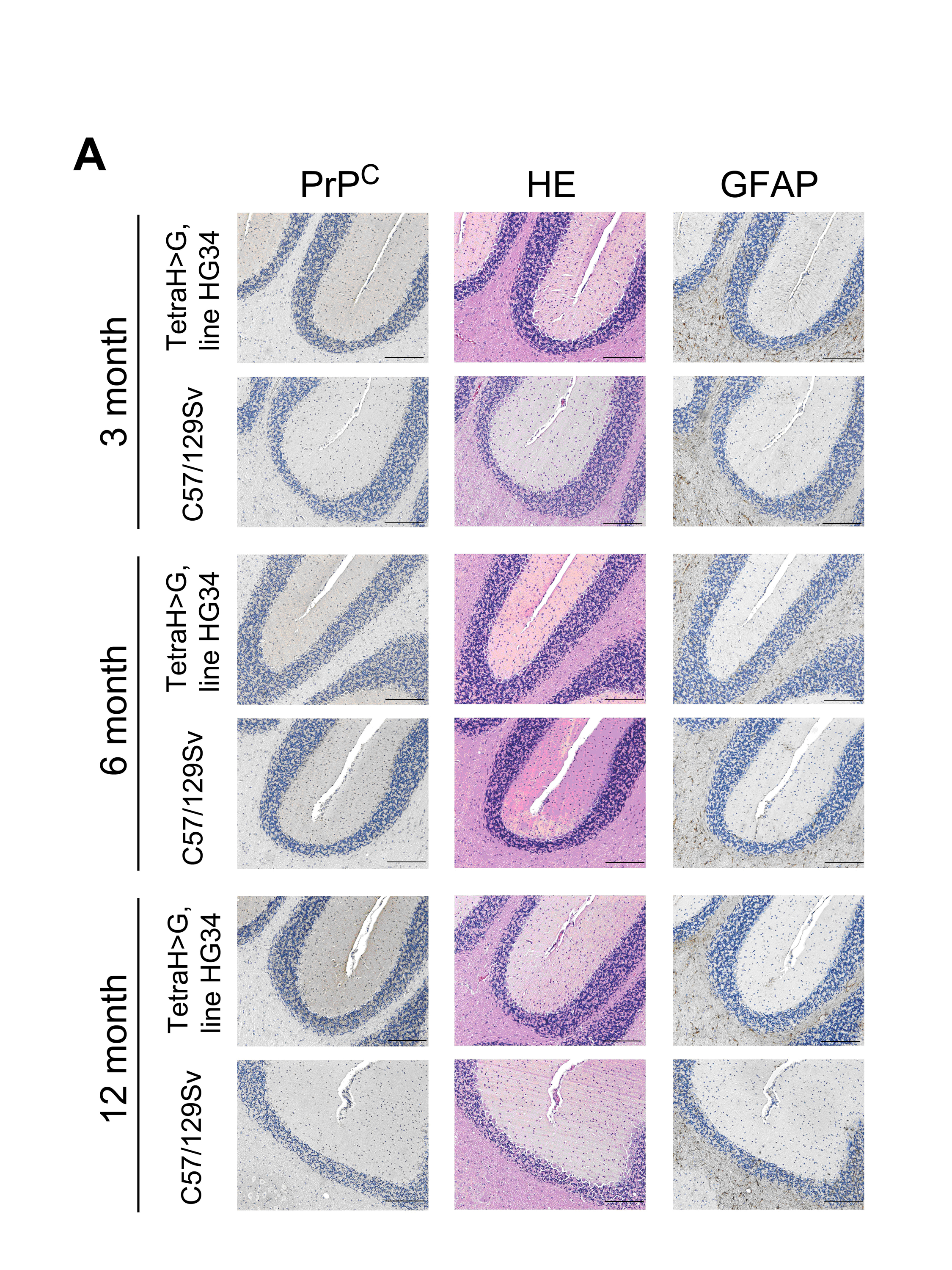

Supplement: S1 Fig — TetraH>G line 34 mice did not develop cerebellar lesions or dysmorphology that distinguished them from non-Tg control wt C57/129Sv mice. (TIF) [file pone.0188989.s001.tif]

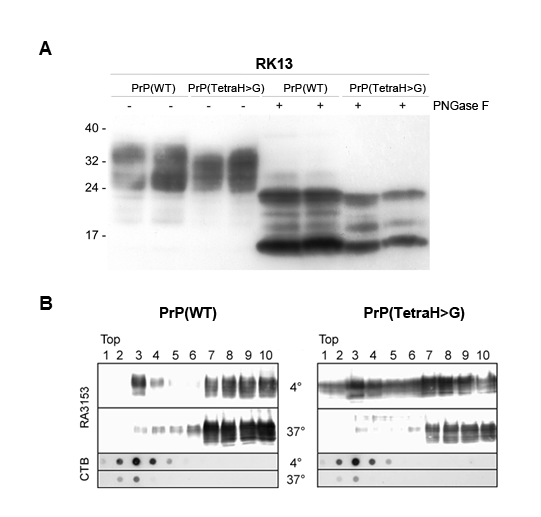

Supplement: S2 Fig — (A) Expression level and glycosylation pattern of wild-type and mutant PrP in RK13 (left panel) respectively, monitored by Western blot analysis using monoclonal antibody 6H4 after SDS-PAGE of whole cell lysates before (-) and after (+) treatment with PNGase F. Molecular weights are indicated on the left (in kDa). (B) Localization of wild-type and mutant PrP to lipid rafts. RK13 cells transfected to express full-length mouse PrP (left panel) or PrP(TetraH>G) (right panel) were cultivated to confluence and then placed on ice or kept at 37°C. Sucrose gradient flotation was done with cell lysates. Aliquots of each fraction collected from the top of the gradient were precipitated, subjected to SDS-PAGE and immunoblot analysis using antibody RA3153. Dot Blot analysis with horseradish-peroxidase conjugated cholera toxin subunit B (Molecular Probes®, Leiden, Netherlands) was performed to detect the lipid rafts containing fractions. (TIF) [file pone.0188989.s002.tif]

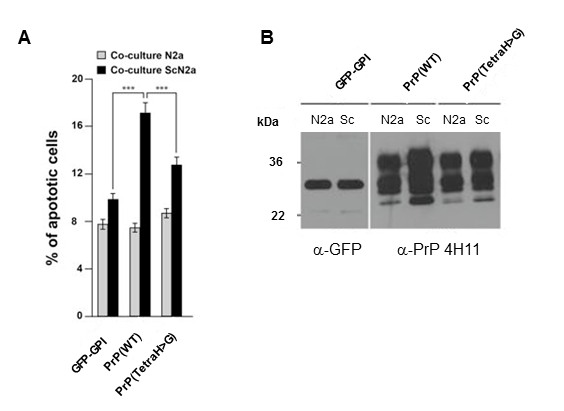

Supplement: S3 Fig — (A) SH-SY5Y cells expressing full-length wild-type PrP (PrP), or PrP(TetraH>G), PrP(H95G) or GPI-coupled GFP (GPI-GFP) were co-cultured with prion-infected (ScN2a) or uninfected neuroblastoma cells (N2a) for 16 h. For quantification of apoptotic cell death, SH-SY5Y cells were fixed, permeabilized and stained for active caspase-3. The percentage of apoptotic cells among transfected cells is shown. (B) Expression of transfected constructs analyzed by Western blotting using the anti-PrP antibody 4H11 or an anti-GFP antibody. Molecular weights (kDa) are given on the left. *** p < 0.0005. (TIF) [file pone.0188989.s003.tif]

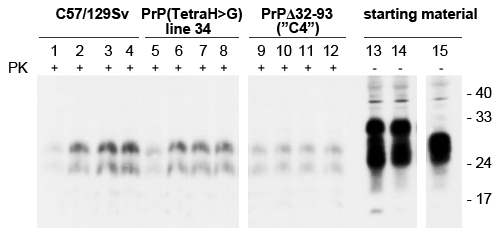

Supplement: S4 Fig — Prior to PMCA, healthy brain homogenates were adjusted to 60 ng PrPC/μl with 10% (wt/vol) PrP0/0 brain homogenate, spiked 1:50 with 10% (wt/vol) RML homogenate and subjected to 10 rounds of PMCA. In vitro generated wt PrPres (C57/129Sv; lanes 1–4), PrPres(TetraH>G) lines 34 (lanes 5–8), as well as PrPΔ32–93 (C4; lanes 9–12) was detected using monoclonal antibody 4H11. In order to rule out degradation of PrPC before PMCA, the corresponding undigested starting material was loaded as control (lane 13: wt; lane 14: PrP(Tetra H>G), line 34, lane 15: C4). Molecular weights are indicated on the right (in kDa). All samples were blotted onto the same membrane and exposed for the same amount of time; irrelevant lanes have been excised at two positions. (TIF) [file pone.0188989.s004.tif]
