# Supplementary material for: Substitutions of PrP N-terminal histidine residues modulate scrapie disease pathogenesis and incubation time in transgenic mice
Source: PLoS One. 2017 Dec 8;12(12):e0188989. doi: 10.1371/journal.pone.0188989 (PMC5722314; doi:10.1371/journal.pone.0188989)
Supplement: S1 Text — (DOC) [file pone.0188989.s005.doc]

**Supporting material and methods**

**Cell transfection**

All cells were cultured as described previously [1]. Transfection of cells with LipofectAMINE 2000 reagent (Invitrogen, Carlsbad, CA, USA) was performed according to the manufacturer´s instructions. Single cell clones derived from the PrP^C^-deficient rabbit kidney epithelial cell line RK13 [2] were co-transfected with vectors pcDNA3.1/Hygro(+) (Invitrogen) and pCINeo-Mowt or pCINeo-MoPrP(TetraH>G) for expressing of full-length mouse wild-type PrP^C^ or PrP(TetraH>G). Single cell clones resistant to hygromycin B (500 μg/ml) were expanded and the level of PrP^C^ expression was monitored by Western Blot analysis of whole cell lysates. Briefly, cells were washed twice with cold PBS, lysed on ice in cold 1 × TNE buffer [150 mM NaCl, 50 mM Tris-HCl (pH 7.5), 5 mM EDTA, and 0.2% (w/v) sarcosyl] and precleared by centrifugation (16,000 × g, 10 min, 4°C). To determine PrP^C^ expression levels in transfected cells, equal amounts of total protein, determined by BCA test (Sigma-Aldrich, St Louis, MO, USA), were separated by sodium dodecyl sulphate polyacrylamide gel electrophoresis and subjected to Western blot analysis using mouse monoclonal anti-prion antibody 4H11 and horseradish peroxidase-conjugated anti-mouse IgG as a secondary antibody.

**Immunofluorescence**

Cells stably expressing mouse full-length wild-type, PrP(TetraH>G) or PrP(H95G) before and after exposure to RML prions were cultivated on 20 mm glass cover slips to 40% confluence and then placed on ice for immunofluorescence staining procedures. Interrupted by two or more washing steps using ice cold PBS, cells were fixed with 3.7% formalin in PBS for 5 min. PrP^Sc^ was revealed specifically by a 5 min pre-treatment with 98% formic acid to remove host PrP^C^ [3]. PK digestion and guanidine hydrochloride (GdnHCl) treatment to remove PrP^C^ and expose PrP^Sc^ were performed as described previously [4]. Formic acid-treated and -untreated cells were permeabilized with cold 1% Triton X-100 for 5 min and then washed with PBS, before blocking with 1% BSA in PBS and subsequent overnight incubation with primary antibody 4H11. Following another two washing steps, Alexa488-labeled goat anti-mouse secondary antibody (Life Technologies, Darmstadt, Germany) was applied under the same conditions. Following further washing steps, cover slips were mounted on glass microscope slides using DAKO Fluorescence mounting medium (DAKO, Hamburg, Germany) and analyzed with an Olympus XX microscope with plan-Apochromat x63/1.40 oil DIC objective coupled with Cell^F software (Olympus, Hamburg, Germany).

**Lipid raft isolation**

RK13 cells [2] stably expressing full-length wild-type or PrP(TetraH>G) were cultivated to confluence in 6 cm dishes and then placed on ice or on a heating block at 37°C, respectively. Cell lysis and sucrose gradient procedures were done as described by Brown and Rose [5]. After two washing steps with PBS, cell lysis was carried out in 2 ml of TNE/TX buffer (20 mM Tris-HCl, pH 7.4, 150 mM NaCl, 1 mM EDTA and 1% Triton X-100) at 4°C or 37°C by homogenizing it ten times through a 26 Gauge-needle. The 2 ml lysate was brought to 40% sucrose by mixing 1:1 with 80% sucrose in TNE buffer, overlaid with 4 ml 35% and 2.5 ml 5% sucrose in TNE buffer and then centrifuged at 180,000 × g for 18 h at 4°C in a Sorvall TH-641 rotor. 1 ml fractions were collected from the top of the gradient. From each fraction 100 μl were precipitated with ice-cold ethanol and prepared for Western blot analysis. For localization of lipid rafts 200 μl of each fraction were dot blotted on a nitrocellulose membrane (Whatman Protran®) and G_M1_ ganglioside detection was done with HRP-conjugated cholera toxin subunit B (Molecular Probes®, Invitrogen, Karlsruhe, Germany). For detection of cellular PrP antibody 4H11 was used.

**Protein misfolding cyclic amplification (PMCA) reactions**

Ten percent (wt/vol) homogenates of mouse brain tissues were prepared according to Saborio et al [6]. The relative levels of PrP^C^ expression in healthy PrP(TetraH>G) and wt brains were determined by Western blot analysis using monoclonal antibody 4H11. Briefly, aliquots of 10% brain homogenates were deglycosylated with PNGase F (New England Biolabs, Frankfurt, Germany) according to the manufacturer´s instructions. Cellular PrP in transgenic and wild-type mouse brains was visualized by enhanced chemiluminescence reaction (GE Healthcare, Freiburg, Germany) and quantified against a dilution series of recombinant mouse PrP (Prionics, Schlieren, Switzerland) using a Diana II luminescence imaging system (Raytest, Straubenhardt, Germany) and the AIDA software package.

For PMCA brain homogenates prepared from healthy wt and transgenic mice were adjusted to 60 ng PrP^C^/μl with 10% (wt/vol) PrP^0/0^ brain homogenate and subsequently spiked 1:50 with brain homogenate extracted from mice *i.c.* infected with RML. Samples of 200 μl were subjected to 10 rounds of PMCA each consisting of 5 × 1 s sonication with an ultrasonic microtip probe at 40 % power setting (Sonopuls 2070, Bandelin, Berlin, Germany) followed by 1 h of incubation at 37 °C. For quantification of proteinase K (PK)-resistant PrP ("PrPres") 20 μl-aliquots of the reaction mixture before and after 10 rounds of PMCA were digested with PK (100 μg/ml) for 1 h at 37 °C and analyzed by Western blotting using 4H11-antibody. PrPres was visualized by enhanced chemiluminescence reaction and evaluated densitometrically as described above. Amplification factors were determined by dividing the densitometric signal of the sample obtained after PMCA by the intensity of the starting material.

**Apoptosis assay**

For co-cultivation experiments, human neuroblastoma SH-SY5Y cells (DSMZ number ACC 209) expressing wt and PrP(TetraH>G), respectively, were grown on glass cover slips and transfected with Lipofectamine (Invitrogen) as described [7, 8]. Two hours after transfection cover slips were transferred into dishes containing a 90% confluent cell layer of either chronically scrapie-infected (ScN2a)[9] or normal N2a cells. 16 h after co-cultivation, SH-SY5Y cells were fixed on glass cover slips with 3.7% paraformaldehyde for 20 min, washed and permeabilized with 0.2% Triton X-100 in PBS for 10 min at room temperature. To analyze apoptotic cell death, fixed cells were incubated with an anti-active caspase 3 antibody (Promega, Germany) overnight at 4 °C, followed by an incubation with an Alexa Fluor® 555 fluorescently secondary antibody (Life Technologies, Darmstadt, Germany) for 1 h at room temperature. Cells were then mounted onto glass slides and examined by fluorescence microscopy using a Zeiss Axiovert 200M microscope (Carl Zeiss, Jena, Germany). The numbers of cells positive for activated caspase 3 or cells with fragmented nuclei out of at least 1000 transfected cells were determined in a blinded manner. To analyse levels of transgene expression, cover slips were removed from the co-culture dishes, lysed, and then normalized for protein content before western blot analysis. Quantifications were based on at least three independent experiments. Data were shown as means ± S.E.. Statistical analysis was performed using Student’s *t* test. P-values are as follows: *** p < 0.0005.
