# Supplementary material for: Substitutions of PrP N-terminal histidine residues modulate scrapie disease pathogenesis and incubation time in transgenic mice
Source: PLoS One. 2017 Dec 8;12(12):e0188989. doi: 10.1371/journal.pone.0188989 (PMC5722314; doi:10.1371/journal.pone.0188989)
Supplement: S2 Text — (DOC) [file pone.0188989.s006.doc]

**Supporting results**

**Performance and cellular localization of PrP with impaired copper binding sites**

In contrast to some PrP alleles with interstitial deletions nullifying copper-binding properties of the OR region but yet resembling mice with a more precise OR region deletion, line 34 TetraH>G mice had no propensity to cerebellar degeneration or dysmorphology (S1 Fig) [10, 11]. Line 34 TetraH>G retained a wt PrP-like insofar as the transgene array could gentically antagonize the action of a patheogenic internally deleted form of PrP (data not shown).

After its synthesis in the endoplasmic reticulum PrP^C^ usually transits through the Golgi to the cell surface where it is bound to the outer membrane of neurons by a C-terminal GPI-anchor [12, 13]. Moreover, GPI-anchored prion protein has been localized at the cell surface within detergent-resistant membrane microdomains that are enriched in cholesterol and sphingolipids and are commonly designated lipid rafts [514]. The association of PrP^C^ with lipid rafts is thought to be a prerequisite for PrP^Sc^ formation [15-21]. Thus, to analyze the cellular localization of PrP with impaired copper binding within the OR region we first performed immunofluorescence staining of cells expressing full-length mouse wt or mutant PrP and found that the mutant proteins localized to the plasma membrane as expected (data not shown). These experiments used RK13 cells (established from the renal tissue of rabbits) expressing robust levels of full-length mouse wt PrP and PrP(TetraH>G), respectively. Western Blot analyses using whole cell lysates from cells expressing mutant PrPs revealed no disparities in glycosylation pattern when compared to corresponding wt controls (S2A Fig). Furthermore, removal of N-linked glycans with PNGase F prior to Western blot analysis resulted in a similar banding pattern of mutated PrP^C^ compared to wt PrP^C^ (S2A Fig), with the minor distinction that a C2-like fragment becomes more emphasized, an apparent point of similarity with the PrP OR region mutant allele denoted S3 [22].

Next, we addressed whether the mutant proteins are correctly inserted in the plasma membrane and reside within lipid rafts. Thus, RK13 cells expressing either full-length wt or mutant PrP were subjected to solubilization with 1% Triton X-100 at 4 °C and 37 °C, respectively. Lipid rafts are resistant to solubilization with cold Triton X-100 and can be isolated by flotation in sucrose gradients, while they tend to be solubilized by Triton X-100 at elevated temperatures. Using this approach, we could confirm that substitution of the histidine residues within the OR region with glycine residues does not prevent the mutated proteins from their correct insertion into the plasma membrane and incorporation into lipid rafts. As shown in S2B Fig, TetraH>G PrP is localized within lipid rafts which refer to fractions 3 and 4 as proven by the presence of the spingolipid GM1. At 37 °C however, solubilization with Triton X-100 drastically reduced the detection of GM1 in fractions 3 and 4, which was paralleled by a dramatic reduction of the PrP signal intensity in the same fractions; this situation was mirrored by WT PrP.

**The PrP(TetraH>G) allele is mildly impaired in mediating the toxic effects of scrapie prions in stably transfected SH-SY5Y cells**

Recent studies demonstrated that binding of β-sheet rich conformers, such as PrP^Sc^ and A β 42, to the N-terminal domain of PrP^C^ induces a switch from neuroprotective to neurotoxic signalling independent of prion propagation [8]. Using an apoptosis assay which is based on the co-cultivation of PrP^C^-expressing cells with scrapie-infected cells that release PrP^Sc^ and infectious prions into the cell culture medium Rambold et al [23] demonstrated that cells expressing PrP devoid of the intrinsically disordered N-terminal domain (PrPΔ27-89) is impaired in mediating the toxic effects of PrP^Sc^. Consequently, we tested whether PrP with all four histidine residues within the octarepeats replaced with glycine is as well impaired in mediating the switch from the pro-survival to pro-apoptotic pathway in the presence of scrapie prions. To this end we co-cultivated SH-SY5Y cells expressing the mutant proteins with scrapie-infected mouse neuroblastoma cells (ScN2a) and determined the extent of apoptotic cell death. Indeed, the ability of both PrP(TetraH>G) was significantly impaired in mediating the toxic effects of PrP^Sc^ compared to SH-SY5Y cells expressing wild-type PrP^C^, as indicated by reduced levels of activated caspase 3 (S3A Fig). Notably, PrP(TetraH>G) were subject to complex glycosylation (S3 B Fig) and presentation at the outer side of the plasma membrane, similarly to wt PrP^C^ (data not shown). Control (i.e. untransfected) SH-SY5Y cells could be co-cultured with ScN2a cells without adverse effects (S3A Fig).

***In vitro* conversion of mutant proteins using protein misfolding cyclic amplification (PMCA)**

According to Saborio et al [6] the structural conversion of PrP^C^ to the aberrant isoform can be modelled *in vitro* using protein misfolding cyclic amplification (PMCA). Thus, brain homogenates extracted from PrP(TetraH>G) mice derived from founder line 34 and corresponding wt control were adjusted to the same amount of PrP^C^ (S4 Fig), spiked 1:50 with RML homogenate, and then subjected to ten alternating rounds of sonication and incubation (corresponding to one cycle of PMCA) in triplicates. After one cycle of PMCA signal intensities corresponding to *in vitro* generated PK-resistant wt PrP (wt PrPres) increased on average by a factor of 5.6 (S4 Fig, compare lane 1 and lanes 2-4). In contrast, the average signal intensity corresponding to PrPres(TetraH>G) increased less strong (factor 3.4) (line 34; S4 Fig, lanes 6-8). Noteworthy, almost no amplification was observed with RML spiked homogenates extracted from "C4" mice expressing PrP devoid of residues 32-93 containing the octarepeats (S4 Fig, lanes 9-12), which fits well to the findings that PrPΔ32-93 mice were still susceptible to scrapie infection, but presented with longer incubation times and approximately 30-fold lower prion titers and PK-resistant PrP than wt mice [11].
