# Supplementary material for: Substitutions of PrP N-terminal histidine residues modulate scrapie disease pathogenesis and incubation time in transgenic mice
Source: PLoS One. 2017 Dec 8;12(12):e0188989. doi: 10.1371/journal.pone.0188989 (PMC5722314; doi:10.1371/journal.pone.0188989)
Supplement: S3 Text — (DOC) [file pone.0188989.s007.doc]

**Supporting references**

1. Lorenz H, Windl O, Kretzschmar HA. Cellular phenotyping of secretory and nuclear prion proteins associated with inherited prion diseases. J Biol Chem 2002; 277: 8508-16. doi: 10.1074/jbc.M110197200 PMID: 11756421

2. McCarthy K, Taylor-Robinson CH, Pillinger SE. Isolation of Rubella Virus from Cases in Britain. Lancet 1963; 2: 593-8. doi: 10.1016/S0140-6736(63)90393-3 PMID: 14050876

3. Kristiansen M, Messenger MJ, Klohn PC, Brandner S, Wadsworth JD, Collinge J, et al. Disease-related prion protein forms aggresomes in neuronal cells leading to caspase activation and apoptosis. J Biol Chem 2005; 280: 38851-61. doi: 10.1074/jbc.M506600200 PMID: 16157591

4. Veith NM, Plattner H, Stuermer CA, Schulz-Schaeffer WJ, Burkle A. Immunolocalisation of PrPSc in scrapie-infected N2a mouse neuroblastoma cells by light and electron microscopy. Eur J Cell Biol 2009; 88: 45-63. doi: 10.1016/j.ejcb.2008.08.001 PMID: 18834644

5. Brown DA, Rose JK. Sorting of GPI-anchored proteins to glycolipid-enriched membrane subdomains during transport to the apical cell surface. Cell 1992; 68: 533-44. doi: 10.1016/0092-8674(92)90189-J PMID: 1531449

6. Saborio GP, Permanne B, Soto C. Sensitive detection of pathological prion protein by cyclic amplification of protein misfolding. Nature 2001; 411: 810-3. doi: 10.1038/35081095 PMID: 11459061

7. Winklhofer KF, Heske J, Heller U, Reintjes A, Muranyi W, Moarefi I, et al. Determinants of the in vivo folding of the prion protein. A bipartite function of helix 1 in folding and aggregation. J Biol Chem 2003; 278: 14961-70. doi: 10.1074/jbc.M209942200 PMID: 12556465

8. Resenberger UK, Harmeier A, Woerner AC, Goodman JL, Muller V, Krishnan R, et al. The cellular prion protein mediates neurotoxic signalling of beta-sheet-rich conformers independent of prion replication. EMBO J 2011; 30: 2057-70. doi: 10.1038/emboj.2011.86 PMID: 21441896

9. Butler DA, Scott MRD, Bockman JM, Borchelt DR, Taraboulos A, Hsiao KK, et al. Scrapie-infected murine neuroblastoma cells produce protease-resistant prion proteins. J Virol 1988; 62: 1558-64. PMID: 3282080

10. Shmerling D, Hegyi I, Fischer M, Blattler T, Brandner S, Götz J, et al. Expression of amino-terminally truncated PrP in the mouse leading to ataxia and specific cerebellar lesions. Cell 1998; 93: 203-14. doi: 10.1016/S0092-8674(00)81572-X PMID: 9568713

11. Flechsig E, Shmerling D, Hegyi I, Raeber AJ, Fischer M, Cozzio A, et al. Prion protein devoid of the octapeptide repeat region restores susceptibility to scrapie in PrP knockout mice. Neuron 2000; 27: 399-408. doi: 10.1016/S0896-6273(00)00046-5 PMID: 10985358

12. Stahl N, Borchelt DR, Hsiao K, Prusiner SB. Scrapie prion protein contains a phosphatidylinositol glycolipid. Cell 1987; 51: 229-40. doi.org/10.1016/0092-8674(87)90150-4 PMID: 2444340

13. Caughey B, Race RE, Ernst D, Buchmeier MJ, Chesebro B. Prion protein biosynthesis in scrapie-infected and uninfected neuroblastoma cells. J Virol 1989; 63: 175-81. PMID: 2562814

14. Pike LJ. Lipid rafts: heterogeneity on the high seas. Biochem J 2004; 378:281-92. doi: 10.1042/BJ20031672 PMID: 14662007

15. Borchelt DR, Taraboulos A, Prusiner SB. Evidence for synthesis of scrapie prion proteins in the endocytic pathway. J Biol Chem 1992; 267: 6188-99. PMID: 1353761

16. Taraboulos A, Scott M, Semenov A, Avrahami D, Laszlo L, Prusiner SB. Cholesterol depletion and modification of COOH-terminal targeting sequence of the prion protein inhibit formation of the scrapie isoform.. J Cell Biol 1995; 129: 121-32. doi: 10.1083/jcb.129.1.121 PMID: 7698979

17. Kaneko K, Vey M, Scott M, Pilkuhn S, Cohen FE, Prusiner SB. COOH-terminal sequence of the cellular prion protein directs subcellular trafficking and controls conversion into the scrapie isoform. Proc Natl Acad Sci U S A 1997; 94: 2333-8. doi: 10.1073/pnas.94.6.233 PMID: 9122195

18. Naslavsky N, Shmeeda H, Friedlander G, Yanai A, Futerman AH, Barenholz Y, et al. Sphingolipid depletion increases formation of the scrapie prion protein in neuroblastoma cells infected with prions. J Biol Chem 1999; 274: 20763-71. doi: 10.1074/jbc.274.30.20763 PMID: 10409615

19. Gilch S, Winklhofer KF, Groschup MH, Nunziante M, Lucassen R, Spielhaupter C, et al. Intracellular re-routing of prion protein prevents propagation of PrP(Sc) and delays onset of prion disease. EMBO J 2001; 20: 3957-66. doi: 10.1093/emboj/20.15.3957 PMID: 11483499

20. Beranger F, Mange A, Goud B, Lehmann S. Stimulation of PrP(C) retrograde transport toward the endoplasmic reticulum increases accumulation of PrP(Sc) in prion-infected cells. J Biol Chem 2002; 277: 38972-7. doi: 10.1074/jbc.M205110200 PMID: 12163492

21. Sanghera N, Pinheiro TJ. Binding of prion protein to lipid membranes and implications for prion conversion. J Mol Biol 2002; 315: 1241-56. doi: 10.1006/jmbi.2001.5322 PMID: 11827491

22. Lau A, McDonald A, Daude N, Mays CE, Walter ED, Aglietti R, et al. Octarepeat region flexibility impacts prion function, endoproteolysis and disease manifestation. EMBO Mol Med 2015; 7: 339-56. doi: 10.15252/emmm.201404588 PMID: 25661904

23. Rambold AS, Muller V, Ron U, Ben-Tal N, Winklhofer KF, Tatzelt J. Stress-protective signalling of prion protein is corrupted by scrapie prions. Embo J 2008; 27: 1974-84. doi: 10.15252/emmm.201404588 PMID: 25661904
